# Temporal changes in DNA methylation and RNA expression in a small song bird: within- and between-tissue comparisons

**DOI:** 10.1186/s12864-020-07329-9

**Published:** 2021-01-07

**Authors:** Melanie Lindner, Irene Verhagen, Heidi M. Viitaniemi, Veronika N. Laine, Marcel E. Visser, Arild Husby, Kees van Oers

**Affiliations:** 1 Department of Animal Ecology, Netherlands Institute of Ecology (NIOO-KNAW), P.O. Box 50, Wageningen, 6700 AB The Netherlands; 2 Chronobiology Unit, Groningen Institute for Evolutionary Life Sciences (GELIFES), University of Groningen, Groningen, The Netherlands; 3 Wageningen University & Research, Wageningen, The Netherlands; 4 Organismal and Evolutionary Biology Research Programme, University of Helsinki, Helsinki, Finland; 5 Institute of Vertebrate Biology, Czech Academy of Sciences, Prague, Czech Republic; 6 Department of Biology, University of Turku, Turku, Finland; 7 Finnish Museum of Natural History, University of Helsinki, Helsinki, Finland; 8 Evolutionary Biology, Department of Ecology and Genetics, Uppsala University, Uppsala, Sweden; 9 Department of Biology, NTNU, Centre for Biodiversity Dynamics, Trondheim, Norway

**Keywords:** DNA methylation, RNA expression, Tissue-specific and tissue-general temporal changes, Accessible and inaccessible tissues, Great tit

## Abstract

**Background:**

DNA methylation is likely a key mechanism regulating changes in gene transcription in traits that show temporal fluctuations in response to environmental conditions. To understand the transcriptional role of DNA methylation we need simultaneous within-individual assessment of methylation changes and gene expression changes over time. Within-individual repeated sampling of tissues, which are essential for trait expression is, however, unfeasible (e.g. specific brain regions, liver and ovary for reproductive timing). Here, we explore to what extend between-individual changes in DNA methylation in a tissue accessible for repeated sampling (red blood cells (RBCs)) reflect such patterns in a tissue unavailable for repeated sampling (liver) and how these DNA methylation patterns are associated with gene expression in such inaccessible tissues (hypothalamus, ovary and liver). For this, 18 great tit (*Parus major*) females were sacrificed at three time points (*n* = 6 per time point) throughout the pre-laying and egg-laying period and their blood, hypothalamus, ovary and liver were sampled.

**Results:**

We simultaneously assessed DNA methylation changes (via reduced representation bisulfite sequencing) and changes in gene expression (via RNA-seq and qPCR) over time. In general, we found a positive correlation between changes in CpG site methylation in RBCs and liver across timepoints. For CpG sites in close proximity to the transcription start site, an increase in RBC methylation over time was associated with a decrease in the expression of the associated gene in the ovary. In contrast, no such association with gene expression was found for CpG site methylation within the gene body or the 10 kb up- and downstream regions adjacent to the gene body.

**Conclusion:**

Temporal changes in DNA methylation are largely tissue-general, indicating that changes in RBC methylation can reflect changes in DNA methylation in other, often less accessible, tissues such as the liver in our case. However, associations between temporal changes in DNA methylation with changes in gene expression are mostly tissue- and genomic location-dependent. The observation that temporal changes in DNA methylation within RBCs can relate to changes in gene expression in less accessible tissues is important for a better understanding of how environmental conditions shape traits that temporally change in expression in wild populations.

**Supplementary Information:**

The online version contains supplementary material available at 10.1186/s12864-020-07329-9.

## Background

Many traits are phenotypically plastic and change with alterations in the environment. This includes circannual traits such as seasonal reproduction in birds: every spring a seasonally breeding female responds to increasing photoperiod and temperature to gradually switch from an inactive state to an active reproductive state such that the specific timing of this response depends on the environmental conditions of the respective year (i.e. the trait is phenotypically plastic) [1]. However, it remains poorly understood how the translation of environmental conditions to a within-individual temporal response in trait value is mediated on the molecular level, i.e. how phenotypic plasticity works.

Epigenetic modifications, like DNA methylation, are known to be able to modulate the expression of phenotypes via an interaction with transcription factors that are required for the initiation of gene transcription [2]. DNA methylation can be highly dynamic in response to environmental signals [3–6] and hence is a candidate for the regulation of transcriptional mechanisms that shape temporally expressed traits [7]. Indeed, changes in DNA methylation were found as a common factor for aging in mammals with a striking tissue-specificity for age related DNA methylation changes [8, 9]. In line with this, DNA methylation regulator genes responded tissue-specifically to acute and chronic stress in chicken (*Gallus gallus*) and hepatic glucocorticoid receptors (GRs) were found to potentially play a critical role in regulating the early-life nutritional stress response of birds [10]. Furthermore, DNA methylation was found to regulate seasonally expressed traits like hibernation of 13-lined ground squirrels (*Ictidomys tridecemlineatus*) [11], photoperiodic diapause timing in a parasitoid insect (*Nasonia vitripennis*) [12], flowering time in plants [13, 14], and timing of reproduction in Siberian hamsters (*Phodopus sungorus*) [5]. The latter study demonstrated that short day length induced a temporal decrease in DNA methylation levels within the promoter region of type III deiodinase (*DIO3*), a gene involved in the photoperiodic regulation of reproduction, and furthermore established a causal link between reduced *DIO3* promoter methylation and gonadal regression via increased transcription of *DIO3* [5].

Most studies on associations between temporal changes in DNA methylation and trait changes are based on between-individual samples, since it is often not feasible to repeatedly sample tissues of biological relevance within the same individual. A more accessible tissue that does allow for repeated within-individual sampling is blood. Avian blood, in contrast to mammalian blood, contains nucleated red blood cells (RBCs), hence more than 90% of the DNA isolated from avian blood originates from erythrocytes [15]. Therefore, only a small amount of avian blood (< 10 μl) is required to isolate sufficient genomic DNA (~ 1 μg) to determine genome-wide DNA methylation profiles via reduced representation bisulfite sequencing (RRBS) [16, 17]. The availability of such a tissue for repeated sampling opens up the possibility to examine within-individual short-term changes in DNA methylation. Indeed, repeated blood sampling of great tit (*Parus major*) females revealed within-individual changes in RBC methylation levels throughout the breeding season that correlated with a female’s reproductive timing [6, 18]. It is, however, unclear to what extent RBC methylation is representative for methylation in (inaccessible) organs. For many phenotypically plastic traits, relevant genes are not expressed in blood, but in more specific tissues. For example, avian timing of breeding requires crucial physiological processes like oviduct development, follicle growth, vitellogenesis and yolk deposition [19]. These processes are regulated by a neuroendocrine cascade, the hypothalamic-pituitary-gonadal-liver axis, which is triggered by environmental information that is received, translated and transduced from the brain [19]. Understanding how transcriptional mechanisms in tissues such as hypothalamus, ovary, and liver that underlie the hypothalamic-pituitary-gonadal-liver axis are regulated throughout the breeding season would give new insights on how females time their breeding. However, repeated sampling in such inaccessible tissues in order to assess within-individual changes in DNA methylation is impossible as it requires sacrificing each individual. Moreover, it would prevent measuring the final trait value, which is the case for timing of breeding where the period of interest starts well ahead of the initiation of egg laying.

Previously, strong correlations have been found between absolute RBC methylation levels and absolute methylation levels in liver, kidney and brain [20, 21]. Therefore, DNA methylation in blood is proposed to be a biomarker for DNA methylation in other tissues. However, it is unknown to what extend *changes* in RBC methylation over time reflect *changes* in DNA methylation over the same time period in other tissues (i.e. tissue-general temporal changes). Here, we explore to what extend temporal changes in DNA methylation are tissue-general or tissue-specific and how tissue-general temporal changes relate to changes in gene expression in the inaccessible tissues of interest. For this, we used 18 captive great tit females that were housed under two controlled temperature environments (three groups of six individuals) that were sacrificed and sampled for RBCs, liver, hypothalamus, and ovary at three time points (six individuals per time point) throughout the pre-laying and egg-laying period. We sequenced the collected tissues to assess DNA methylation levels (RBCs, liver) together with candidate gene (liver, using individual qPCR data) and genome-wide (hypothalamus, ovary and liver, using RNA-seq data of pooled individuals) expression profiles. Our aim was to explore to what extent (i) changes in DNA methylation in RBCs and liver are tissue-general or tissue specific, (ii) changes in liver DNA methylation correlate with changes in the expression of candidate genes within liver, and (iii) changes in RBC and liver methylation reflect changes in genome-wide gene expression in a tissue-general or tissue-specific manner in the hypothalamus, ovary and liver. Potentially, the presence of tissue-general temporal changes in DNA methylation that cause a predictable change in gene expression in inaccessible tissues, will open up the possibility to monitor how environmental conditions affect temporally expressed traits via repeated blood sampling, even in wild populations.

## Results

### Exploration of Reduced Representation Bisulfite Sequencing (RRBS) and RNAseq data sets

Using hierarchical clustering and principal component analysis (PCA) on methylation information from both RBC and liver, samples clustered strongly by tissue (Additional files 1 and 2; Figs. S1 and S2). Within the respective tissue, samples did not cluster by temperature environment or by sampling time point, but some samples clustered by family (Additional files 3, 4, 5 and 6; Figs. S3-S6). We detected one outlier within the RBC samples that remained in the analysis (Additional files 3 and 5; Figs. S3 and S5 but see Additional file 7; Fig. S7 for a PCA excluding the outlier). An exploratory analysis of the RNAseq expression data is presented in [22].

### Tissue-general and tissue-specific changes in DNA methylation between red blood cells and liver

**Fig. 1:**
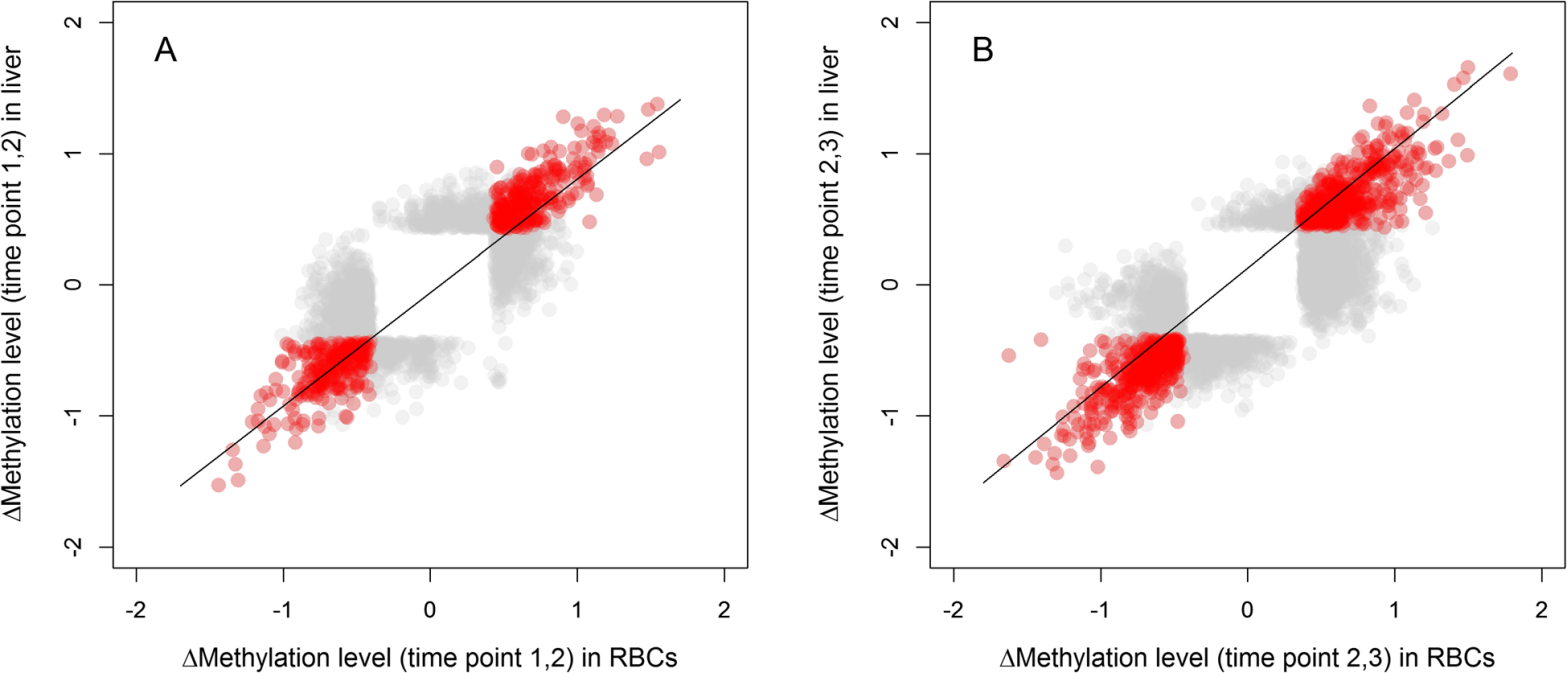
Correlation between CpG sites in RBCs and liver data that show a significant change in methylation for Δ_1,2_ (**a**) and Δ_2,3_ (**b**). Methylation change is visualized as the normalized change (z-scores). We depict sites that significantly change in methylation in both tissues (tissue-general change) in red (*n* = 537 for Δ_1,2_ and 853 for Δ_2,3_) or in one of the tissues (tissue-specific change) in grey (*n* = 1840 for Δ_1,2_ and 3081 for Δ_2,3_). We applied transparency because of the high number of overlapping data points. Line is the regression line

Of the 302,647 CpG sites that were covered by both the RBC and liver data (Additional file 8; Table S1), 2377 CpG sites showed a significant change in methylation between time point 1 and 2 (Δ_1,2_) and 3934 CpG sites changed significantly between time point 2 and 3 (Δ_2,3_) (Additional files 9 and 10; Tables S2 and S3). Methylation changes over time in RBCs showed an overall strong correlation with methylation changes over time in liver for both Δ_1,2_ (*r* = 0.77, df = 2375, *p* < 0.0001, Fig. 1a) and for Δ_2,3_ (*r* = 0.75, df = 3932, *p* < 0.0001, Fig. 1b), when including both the differentially methylated sites (DMS) changing in a tissue-specific way (i.e. only in RBCs or in liver) and DMS changing in a tissue-general way (i.e. in both RBCs and in liver).

**Fig. 2:**
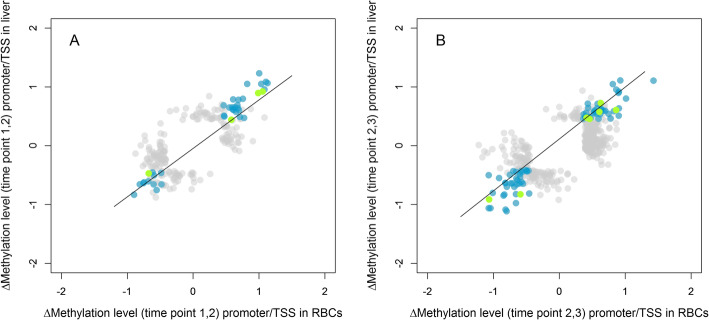
Correlation between the change in methylation of CpG sites in promoter and TSS regions in RBC data with the change in methylation of those in liver data that showed a significant change in methylation for Δ_1,2_ (**a**) and Δ_2,3_ (**b**). Methylation changes are visualized as normalized changes (z-scores). Sites that change significantly in methylation in both tissues (tissue-general change) in promoter and TSS regions are shown in blue (*n* = 38 for Δ_1,2_ and 77 for Δ_2,3_) and green (*n* = 4 for Δ_1,2_ and 7 for Δ_2,3_), respectively. Sites that change significantly in methylation in one of the tissues (tissue-specific change), independent of gene region, are shown in grey (*n* = 287 for Δ_1,2_ and 606 for Δ_2,3_). We applied transparency because of the high number of overlapping data points. Line is the regression line

Out of the 302,647 CpG sites covered by both the RBC and liver data, 108,298 were situated within promoter regions (2000 bp upstream – 200 bp downstream of the annotated gene start). Of these, 221 CpGs were differentially methylated in at least one of these tissues for Δ_1,2_ and 457 CpG sites for Δ_2,3_. The temporal change in methylation of these CpGs in RBCs, was strongly correlated with the temporal change in methylation in liver for both Δ_1,2_ (*r* = 0.74, *n* = 219, *p* < 0.0001, Fig. 2a) and Δ_2,3_ (*r* = 0.70, df = 455, *p* < 0.0001, Fig. 2b), when including DMS that changed in a tissue-specific manner with DMS that changed in a tissue-general manner.

When focusing on the 41,591 CpG sites that were situated near the transcription start site (TSS region, 300 bp upstream – 50 bp downstream of the annotated gene start site) of a gene and covered by both the RBC and liver data, 24 CpG sites showed a significant change over time for Δ_1,2_ and 65 sites for Δ_2,3_ in at least one tissue. Also, when focusing on DMS in the TSS region, the change in methylation in RBCs showed a strong correlation with the change in methylation of these same sites in liver for both Δ_1,2_ (*r* = 0.71, df = 22 *p* = 0.0001, Fig. 2a) and Δ_2,3_ (*r* = 0.62, *n* = 63, *p* < 0.0001, Fig. 2b), when combining DMS that changed in a tissue-specific manner with DMS that changed in a tissue-general manner.

Overall, the number of DMS detected in RBCs was higher compared to the number detected in liver. Also, the number of DMS detected between time points two and three (Δ_2,3_) was higher compared to Δ_1,2_ (Additional file 11; Table S4).

#### Gene ontology analyses

In total 3350 unique great tit genes (Additional file 12; Table S5) were covered when including all DMS (those that changed in a tissue-specific and a tissue-general manner) that were situated in the gene body, 10 kb up- and the 10 kb downstream region (Fig. 1), promoter region or the TSS region (Fig. 2). When including only DMS that changed in a tissue-general manner (in both RBC and in liver), 1153 unique great tit genes were covered (Additional file 12; Table S5), whereas DMS that changed in only one tissue, covered 2352 unique great tit genes for RBCs and 1408 for liver (Additional file 12; Table S5). Using the human gene ontology (GO) database, we found 16 and 28 significant GO terms associated with the genes related to DMS that change in a tissue-general manner and tissue specific manner, respectively (Additional file 13; Table S6). These include four significant GO terms; ‘JAK-STAT signaling pathway’, ‘synaptic vesicle cycle’, ‘carbohydrate digestion and absorption’ and ‘spinocerebellar ataxia’ (Additional file 13; Table S6). Although some of the identified GO terms such as ‘positive regulation of hormone secretion’ and ‘positive regulation of peptide hormone secretion’ potentially have a role in timing of breeding, overall the GO and KEGG terms related to a wide range of functions (Additional file 13; Table S6). Performing GO analyses on sets of genes where DMS were located in the TSS region did not result in any significantly enriched GO or KEGG terms.

### Correlation between change in methylation and candidate gene expression in liver

For the candidate genes, the number of CpG sites with ≥10x coverage ranged between 3 and 15 in the TSS region (*n* = 5) and 6–54 per gene in promoter regions (*n* = 7, Additional file 14; Table S7). No significant correlations were found between the change in DNA methylation over time in CpG sites within a candidate gene and the change in RNA gene expression over time (for both Δ_1,2_ and Δ_2,3_). This was true, when taking into account those CpG sites that were situated within regions known to associate with gene expression in the great tit: in TSS regions or within promoter regions (Additional file 15; Table S8, Additional files 16, 17, 18 and 19; Figs. S8-S11).

### Genome-wide associations between changes in methylation and gene expression

**Fig. 3:**
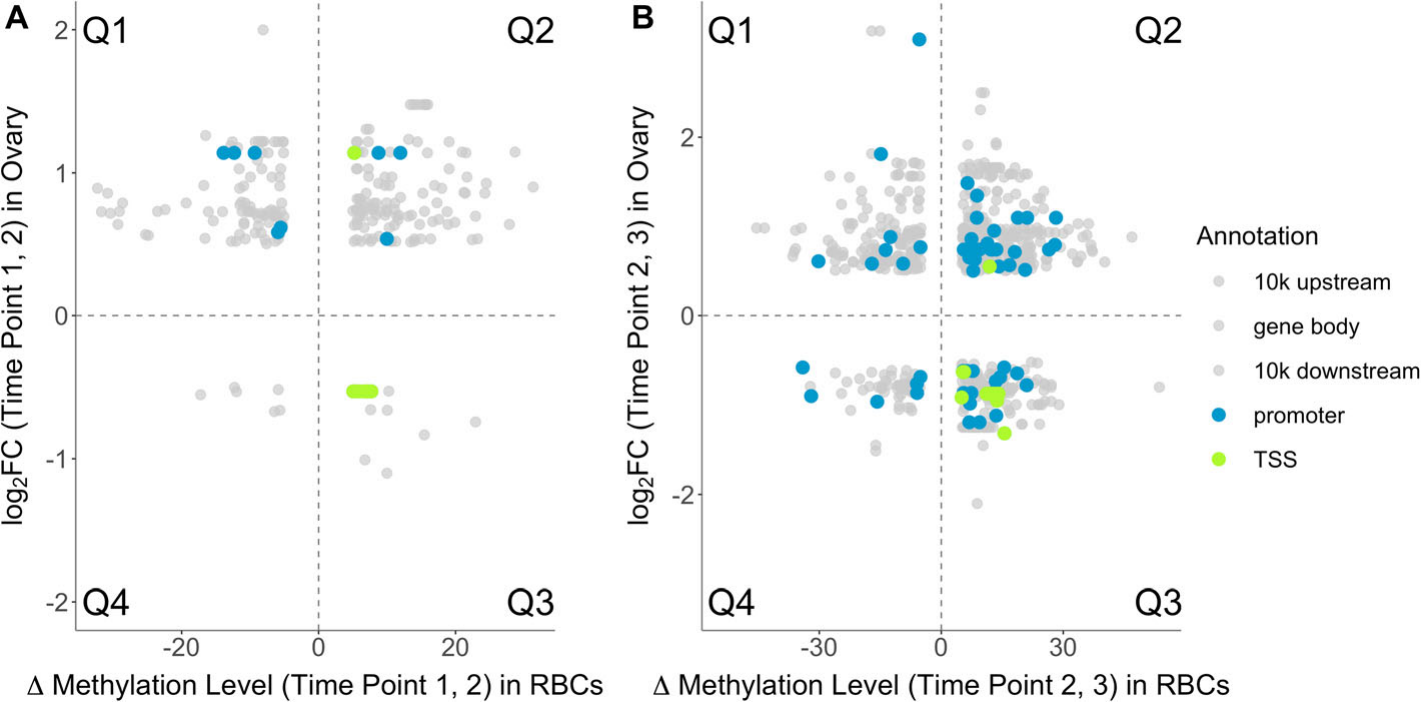
Log_2_ foldchange (log_2_ FC) for the expression of genes in ovary in relation to change in methylation level of a CpG site in RBCs within the TSS region (green), promoter region (blue) or 10 kb up- and downstream region and gene body (all grey) of that gene for Δ_1,2_ (**a**) and Δ_2,3_ (**b**). See Additional files 37 and 38; Tables S18 and S19 for the number of sites and genes for Δ_1,2_ (**a**) and Δ_2,3_ (**b**), respectively. The four quadrants (see ‘Methods’) are separated by dotted lines and labeled as ‘Q1-Q4’. Transparency is applied to the grey data points such that the area of overlap between plots appears darker

To assess the association between changes in methylation and changes in gene expression, we analyzed 297,916 CpG sites that were covered by the RBC data and 529,717 CpG sites that were covered by the liver data. We identified 2256 CpG sites present in the RBC data (Additional file 21; Table S10) and 243 CpG sites in the liver data (Additional file 20; Table S9) that significantly varied in their methylation levels across all time points (i.e. not any particular comparison between time-points). Based on the differential gene expression analysis reported in [22], the expression of 63 genes in hypothalamus (Additional file 22; Table S11), 1073 genes in ovary (Additional file 23; Table S12) and 143 genes in liver (Additional file 24; Table S13) changed significantly (see ‘Methods’ for details) across the time points (*n* = 2 pools per time point with *n* = 3 females per pool). We then analyzed how changes in methylation were associated to changes in gene expression for different tissue comparisons, namely (a) how changes in liver methylation related to the change in liver gene expression, and how changes in RBC methylation related to gene expression change in (b) liver, (c) ovary, and (d) hypothalamus (Additional files 25, 26, 27, 28, 29, 30, 31 and 32; Figs. S12-S19 for all tissue comparisons). Associations between a change in gene expression and a change in CpG site methylation within the gene body, 10 kb up-or downstream region, and promoter region were randomly distributed across all four quadrants (Q1-Q4, see ‘Methods’ for details) without an enrichment for the quadrants with the expected negative relationship between methylation change and gene expression change (i.e. Q1 and Q3, Fig. 3 and Additional files 25, 26, 27, 28, 29, 30, 31 and 32; Figs. S12-S19) irrespective of the tissue comparison (a-d). In contrast, associations within the TSS region were exclusively located within the expected quadrants (Q1 and Q3) when comparing (a) the change in liver methylation to the change in liver gene expression, (b) the change in RBC methylation related to the change in liver gene expression and (d) the change in RBC methylation related to the change in hypothalamus gene expression (Additional files 25, 26, 27, 28, 29, 30, 31 and 32; Figs. S12-S19), although the number of associations for the change in gene expression and change in CpG site methylation was limited (max. four associations per tissue comparison). When comparing (c) the change in RBC methylation in the TSS region with changes in gene expression in ovary, associations in Q1 or Q3 were overrepresented between time point 2 and 3 when compared to associations within the 10 kb downstream region, where we did not expect this effect a priori (Fisher’s Exact Test: *p* = 0.001, Fig. 3b). We found a non-significant trend in the same direction for the change between time point 1 and 2 (Fisher’s Exact Test: *p* = 0.11, Fig. 3a). The genes, the number of associated CpG sites, and the number of associations within quadrants Q1 or Q3 and within quadrants Q2 or Q4 are listed for each combination of comparison (a-d), time contrast (Δ_1,2_ and Δ_2,3_) and genomic location in Additional files 33, 34, 35, 36, 37, 38, 39 and 40; Tables S14-S21.

## Discussion

Evidence that blood-derived measurements of DNA methylation can function as a proxy for DNA methylation values in other tissues is growing [20, 21]. It is unclear though, whether this can be generalized to the context of temporal changes in methylation [23]. Especially in an ecological context, it is currently unknown to what extent temporal changes in DNA methylation are established in a tissue-general or tissue-specific manner and to what extent possible tissue-general changes in DNA methylation are associated with changes in gene expression in various tissues. Here, we explored whether DNA methylation changes over time were tissue-specific or tissue-general (based on change in methylation in RBCs and liver) and how changes in DNA methylation were associated with changes in gene expression of some target tissues unavailable for repeated sampling (hypothalamus, ovary and liver). We found that methylation changes in DMS covered by RBC and liver data acted in parallel. This was true for sites that were situated throughout the whole genome and for sites within regions of the genome where we expect an association between methylation changes and changes in gene expression, i.e. within the promoter or TSS region of annotated genes [24]. For a set of seven candidate genes related to timing of reproduction, we found no correlation between the change in DNA methylation in liver data and the change in gene expression in liver tissue over time. Genome-wide, we found an expected TSS region-specific correlation between an increase in CpG site methylation and a decrease in expression of the associated gene in the ovary. As expected, we found no such association between changes in DNA methylation and expression changes of the respective gene when the site was situated in the gene body or in the 10 kb up- or 10 kb downstream regions, irrespective of which tissues were compared.

Here, we suggest and discuss four possible groups of DMS that categorize how DNA methylation changes over time can differ across tissues and how these changes are associated to differences in changes in gene expression across tissues. The first two groups contain DMS showing a tissue-specific change in DNA methylation that correlates with a change in gene expression in (1) a tissue-specific or (2) tissue-general manner. These groups cannot be used as biomarkers for temporally expressed traits, because of their tissue-specific change in methylation and/or gene expression. Although there is a growing body of studies investigating tissue-specific methylation, these studies are mostly in relation to aging and diseases [25–28]. Further, these studies often do not elucidate the mechanism(s) by which methylation changes and variation in methylation changes across tissues are induced or the functional consequence. It is likely that the (de)methylation mechanism underlying these tissue-specific changes are also tissue-specific. There is some evidence that methylation patterns in tissues are more similar when these tissues are derived from, for example, the same germ layer [29] and that the rate of cell division contributes to tissue-specific methylation profiles [30]. However, whether this relates to tissues-specific changes in methylation, remains to be established.

The other two groups are DMS showing a tissue-general change (Figs. 1 and 2) that correlates with a change in gene expression in (3) a tissue-specific or (4) a tissue-general manner. Both groups can potentially be used as biomarkers for temporally expressed traits, because they change in a similar way across tissues (or at least here, in RBCs and liver) and extrapolation from one tissue to other tissues may be possible. Both groups open up the potential for RBC methylation to be predictive of gene expression changes in other tissues to some extent. However, the universality of this link remains to be established. DMS within group 4 could be mediated by a general increase in body-wide DNA methyltransferase activity, catalysing DNA methylation and preserving methylation after cell division in a tissue-general manner. DMS within group 3 could, for example, be mediated by an environmentally caused release of hormones with system-wide effects, which may have common effects on DNA methylation across tissues, but that differ in magnitude [31]. An example of such a common effect is the activation of the glucocorticoid receptor (*GR*) gene. When stress activates the hypothalamic-pituitary-adrenal axis, cortisol is globally increased. Although GR binding sites show tissue-specificity, their activation is shown across tissues [32]. As such, activation of *GR* may lead to epigenetic changes across tissues, as shown in both humans and rodents [33, 34]. In line with our findings, we *hypothesize* that DMS within the TSS region that are hypomethylated in RBCs could be hypomethylated in a tissue-general manner, but are likely only functional (causing gene expression changes) in the ovary, where the tissue-specific process is performed and inactivated by regulatory mechanisms other than DNA methylation in RBCs where the process is not expressed [35–37]. Here, we hypothesize about a link between tissue-general changes in DNA methylation and tissue-specific changes in gene expression, but our experimental set-up does not allow for strong conclusions and more targeted experiments are needed to follow up on this hypothesis.

Further, it is important to realize that certain tissues, like the brain, liver and ovary, play key roles in traits such as timing of breeding and stress responsiveness, and could have very specific signalling pathways, whereas others are common across tissues [31]. Additionally, in complex tissues, epigenetic mechanisms also differ according to tissue regions, sub-tissue regions, and cell types, as shown previously in human brain [29, 38]. Thus, even though methylation changes in RBCs could potentially predict a part of the methylation change in other tissues, results from epigenetic studies in peripheral blood have to be interpreted with great care with regard to their reflection of epigenetic patterns in highly heterogeneous tissues.

Exploring whether genes carrying DMS that show either a tissue-specific or tissue-general change in the different genomic locations are associated with certain functional groups or GO terms (related to timing of breeding), resulted in several GO terms related to a wide range of biological processes. However, for most of the sites that changed in methylation level in both RBC and liver and most of the sites in the TSS region, no GO terms and pathways were found. Although a small gene set could result into significantly enriched GO terms when they are associated to the same GO terms, the limited number of genes with DMS in the TSS region in this study did not. Also, we found no GO term clearly pointing towards timing of breeding. However, as humans do not reproduce seasonally these human-based ontologies might not include GO terms of functional relevance for species that have a seasonally regulated reproduction.

We also investigated whether changes in RBC methylation correlate with individual gene or genome-wide gene expression changes in other tissues. We found no correlations between the change in CpG site methylation and the change in RNA expression between time points for a set of candidate genes. The genes we analysed, irrespective of whether they were used as a reference gene (*PRCKA*, *RPL19*, *SDHA*) or gene of interest (*HSPB1*, *GR*, *MR*) were expressed very stably over time [39]. As such, it might not be surprising to not find a correlation between the change in methylation and expression for these specific genes. Previous studies in great tits have shown a negative association between TSS region methylation in RBCs and associated gene expression in the brain [21, 24] and found that hypomethylation of CpG sites in the TSS region, which is associated with increased expression, is enriched in genes with functional classes that relate directly to processes specific to the tissue type [21]. Genome-wide, we find a similar trend, in which CpG site hypermethylation within the TSS region in RBCs was predominantly associated with a decrease in the expression of the respective gene, most pronouncedly for the ovary. As expected, no specific trend was found in the 10 kb up- and 10bk downstream region and the gene body, which confirms the lack of association between DNA methylation and gene expression for these regions [24]. In contrast to other studies [21, 24, 40], we did not find a negative correlation between absolute levels of promoter DNA methylation and gene expression, but we have to emphasize here that these studies did not investigate the relationship between the change in DNA methylation and the change in gene expression. This poses the question about how to define the region where gene transcription is initiated and where DNA methylation changes indeed affect gene expression.

We emphasize that the time points and tissues in this study were chosen in relation to timing of breeding, and to explore its underlying molecular mechanisms elsewhere [6, 22, 39]. RBCs are likely to have a limited biological function with regard to complex traits like timing of breeding, since the genes directly responsible for biological functions in this context are expressed in tissues within the hypothalamic-pituitary-gonadal-liver axis, which regulates gonadal function and ultimately egg-laying. Recent studies in great tits, found temporal variation in genome-wide DNA methylation in RBCs collected throughout the breeding season [6] and a correlation between changes in DNA methylation levels and a female’s reproductive timing [18]. The CpG sites in these studies that show a time, treatment or reproductive timing-specific response in DNA methylation are of interest for understanding to what extent DNA methylation acts as a mechanism that translates environmental signals into a phenotypic response, e.g. timing of breeding. However, whether changes in RBC methylation reflect changes in other tissues and how these changes are reflected in gene expression changes in various tissues is not clear. Regardless of the overall strong correlation between methylation change in RBCs and liver needs to be interpreted carefully as this does not imply that RBC derived methylation can always be used as a proxy for methylation patterns in other tissues. This is, because DMS underlying this association include both DMS that change in a tissue-specific and DMS that change in a tissue-general manner (Fig. 1), indicating that both common and unique epigenetic alterations within tissues likely reflect differential functions. Despite the fact that many DMS are tissue-specific and cannot be used as biomarkers for methylation change in other tissues, there is a potential for methylation patterns in RBCs to be informative for a proportion of the temporal changes in methylation patterns in liver.

Although we sampled tissues from individuals at three different time points, these are not within-individual repeated measures as opposed to another study in the same birds using repeated RBC sampling [6]. It is impossible to repeatedly sample tissues like the brain or ovary, and it is highly challenging or even impossible for liver. Here, we thus used a between-individual approach as a proxy of within-individual sampling and acknowledge that we cannot separate between- and within-individual effects. In great tits, however, CpG site methylation in RBCs changes throughout the breeding season within individuals [6] and here we find that DNA methylation changes throughout this period in RBCs and liver based on between-individual samples in a similar way. As such, the time effect on DNA methylation throughout this period seems strong enough to be detected albeit possible between-individual effects resulting from using samples of different individuals at each sampling time.

Unfortunately, we were not able to look at the associations between gene expression and methylation changes in other candidate genes [36] that have been shown to be key in reproductive functioning, as CpG sites within those genes did not have ≥10x coverage for all samples in the RRBS data. Similarly, a limited number of sites was available for the correlation analyses between CpG site methylation and gene expression in a genome-wide approach, especially for CpG site within the TSS regions, as these only span 350pb. Further, the RRBS data was based on individuals, whereas the RNA-seq data originated from pooled samples [22]. The number of pools for the early selection line used in the current study was limited (*n* = 6), and hence, we used genes identified as differently expressed over time (i.e. genes with a time effect and no line effect) in hypothalamus, ovary and liver from the study that used individuals from both selection lines [22]. We calculated the change in methylation level based on samples of individuals and the log2Fold-change in gene expression level based on pooled samples. As described in [22], most differentially expressed genes over time were found in the ovary, while numbers of differentially expressed genes were lower in hypothalamus and liver. As such, we only had enough data points to test for an association between the change in RBC methylation and change in gene expression in the ovary. For the other tissue comparisons (especially liver-liver, RBCs-liver, and RBCs-hypothalamus) there were too few data points (0–4 CpG sites) in the TSS region in comparison to the other genomic locations (gene body, 10 kb upstream and downstream region) to draw any conclusions. Furthermore, CpG sites in the TSS region show a lower methylation level in general than sites within other genomic locations and even low methylation levels (about 20%) within the TSS region were associated to downregulation of the associated gene which was not found for sites within other genomic locations [24]. Thus, analysing sites within the TSS region for differential methylation, together with sites in the other genomic locations might cause a biological relevant change in methylation within the TSS region to appear statistically insignificant based on the high number of tests performed with sites in genomic locations that show changes in higher magnitudes.

## Conclusions

In general, we found that temporal changes in DNA methylation correlate well between tissues. This indicates that the mechanisms underlying these DNA methylation changes over time do not act in the target tissues only, but may be general throughout the body for a large proportion of sites and likely have a genetic basis [41]. However, the vast majority of changes in DNA methylation were not associated with gene expression changes in target tissues in a predictable way. Predictable changes were only present for sites in the TSS region, albeit supported by a few data points only. This shows that general patterns of DNA methylation in any tissue cannot be taken as predictive values for gene expression changes in other tissues and the effects of methylation changes are likely very targeted. Nevertheless, this study provides insights into temporal changes in methylation across tissues and how these changes relate to changes in gene expression. This highlights the importance for distinguishing between tissue-specific and tissue-general changes in DNA methylation, as the latter can be informative for changes in DNA methylation in inaccessible tissues and possibly changes in gene expression. As such, a better understanding of these tissue-general patterns opens up the possibility to monitor the effect of environmental conditions on temporally plastic traits via repeated blood sampling, even in wild populations.

## Methods

### Sample origin

The 18 females used in this study were part of a larger study. For a detailed description of the experimental setup and sampling of that larger study see [39]. In short, 36 great tit pairs (18 *early* selection line pairs and 18 *late* selection line pairs in their second calendar year) that constitute the F_2_-generation of lines artificially selected for early and late timing of breeding [42, 43], were housed in 36 climate-controlled aviaries (2 m × 2 m × 2.25 m) at the Netherlands Institute of Ecology (NIOO-KNAW). Every climate-controlled aviary contained three nest boxes, a perch, fake tree, a food and water tray and bedding of wood chips. All great tits in this study decent from a wild long-term study population at the Hoge Veluwe National Park, The Netherlands (52°02′07″ N, 5°51′32″ E). Per selection line, pairs were formed randomly, but avoiding sibling pairings. Birds were subjected to a photoperiod mimicking the natural photoperiod and two contrasting temperature environments mimicking a cold spring (2013) and a warm spring (2014) in the Netherlands. Temperatures changed every hour to follow as closely as possible the observed hourly temperatures in these years. The combination of selection line and temperature environment resulted in four groups of *n* = 9 pairs within each group: ‘early-warm’, ‘early-cold’, ‘late-warm’ and ‘late-cold’. Within selection line, birds were randomly assigned to an aviary, but temperature environment (warm or cold) would alternate every aviary and selection line (early or late) every two aviaries, resulting in the order ‘early-warm’, ‘early-cold’, ‘late-warm’, and ‘late-cold’ throughout the 36 climate-controlled aviaries. Birds were fed ad libitum with food sources reported elsewhere [44] and had water available for drinking and bathing.

Although great tits normally only have one reproductive season per year, the pairs included in this larger study were induced to undergo two reproductive seasons [for details see 32]. In short, in the first breeding event from January until July, individuals were blood sampled bi-weekly [6, 16], and laying dates were obtained. Then, birds went through a period of short-day length (L:D 10:14) and low temperatures (10 °C) to induce gonadal regression and to make them photoreceptive and temperature sensitive again. Subsequently, birds received the same temperatures and photoperiods as in the first breeding event to induce a second breeding event that was initiated in autumn (September until November). The 36 pairs were divided into three groups taking into account the females’ laying dates in the first breeding season [39], and sacrificed at three time points throughout this second breeding event (see below).

### Tissue collection and preparation

Based on the reproductive behavior during the first breeding event, three sampling time points throughout the second breeding season were chosen: (1) October 7 (resembling March 7) when photoperiod exceeded 11 h, which is necessary to initiate gonadal maturation [45], (2) October 28 (resembling March 30) when nest building occurred in the first breeding season, but prior to laying and (3) November 18 (resembling April 20) when 25% of the females had initiated egg laying in the first breeding event. Per time point both sexes of one group (*n* = 12 pairs) were sacrificed, although we focus on females only in the current study [39]. To sample birds, they were caught per pair from their aviary between 9:00 AM and 13:15 PM, taken to the dissection room and weighed (body mass (g) ± s.e.m. (range), males: 18.3 ± 0.2 (16.3–20.5) and females: 17.03 ± 0.17 (15.0–20.0)). Subsequently, taking into account the least amount of stress and highest sample quality, birds were anaesthetized deeply through inhalation of Isoflurane (vaporizer setting 2.5–3.0%), during which a blood sample (300 μl) was taken. RBCs were separated from plasma by centrifuging (10 min at 14,000 rpm) and stored in Queens buffer at room temperature before further analysis (see ‘Reduced representation bisulfite sequencing (RRBS)’ below). Following decapitation, tissues, including brain, ovary and liver were dissected and stored in − 80 °C until further processing. At a later stage, the hypothalamus, being the center for integration, transduction and translation of environmental cues, was isolated from the rest of the brain and, until further processing, stored in − 80 °C.

The samples that we used in this study are from the early selection line females in the second (autumn) breeding season (*n* = 18, with 6 females per sampling time point) because during this sampling event blood, hypothalamus, ovary and liver where collected as opposed to the first breeding event where only blood was sampled.

### Reduced Representation Bisulfite Sequencing (RRBS)

We extracted DNA from RBCs stored in 250 μl Queens buffer (with approximately 10–20 μl of RBCs per 1 ml) using the DNeasy kit (Qiagen) and from 25 mg liver with the MagAttract kit (Qiagen) according to manufacturer’s protocol. To produce Reduced Representation Bisulfite Sequencing libraries, the preparation protocol according to manufacturer’s protocol (Illumina) was used with some changes [46]. Briefly, samples were digested using the restriction enzyme MspI and the resulting DNA fragments of various size were subsequently bisulfite treated, which converts un-methylated cytosine bases into uracil bases, whereas methylated cytosine bases are resistant to the treatment. Fragmented and bi-sulfite treated DNA was then end-repaired with DNA polymerase I and A-overhangs were added to the 3′ ends of each fragment for adapter ligation. Individual sample libraries were barcoded using standard Illumina adapters. Libraries were purified, size selected with Ampure XP beads (Beckman Coulter) and concentrations were determined by quantitative polymerase chain reaction (qPCR). This selection yielded a fragment size range of approximately 30–180 base pairs, with a mean of 85. Six libraries were pooled into the same sequencing lane (Additional file 41; Table S22). Each pool was sequenced 100 bp single end (Additional file 41; Table S22) on a HiSeq2500 sequencer with a HiSeq SBS sequencing kit version 4 (Illumina). Sequencing was conducted in two separate HiSeq runs to yield enough coverage per sample. An internal positive control (PhiX) was used to obtain reliable sequence generation in the sequencing processing and the PhiX reads and adapters were removed before data analysis. Library preparation and sequencing were performed at the SciLife Lab, Uppsala University, Sweden.

#### Sequence read quality and alignment

Sequencing read quality was investigated with the FastQC 0.11.7 quality control tool [47]. Low quality bases as well as Illumina adapter contamination resulting from read-through of short fragments were trimmed using Trim Galore! v0.4.4 [48] with default parameters under the *–rrbs* mode. This mode disregards the first five base pairs in the 5′ to reduce calling of false positive methylation as a result of bisulfite treatment. Each sample’s reads from both of the sequencing runs were combined together for alignment. Trimmed sequencing reads were aligned against a bisulfite converted version of the *Parus major* reference genome v1.1 (https://www.ncbi.nlm.nih.gov/assembly/GCF_001522545.2) using Bismark 0.19.1 (Bioinformatics Group. Babraham Institute) aligner in *rrbs* mode. The reference genome contains all assembled chromosomes as well as all scaffolds. After alignment and CpG site calling we selected the sites with a minimum coverage of 10x across all samples within a tissue (RBCs and liver) for further analyses. We calculated the methylation proportion for a site in the respective sample as the proportion of methylated counts relative to the total read counts. As we were interested in sites that change over time, we excluded all sites that showed a methylation proportion of either zero or one across all samples from downstream analyses.

#### Gene annotation

**Fig. 4:**
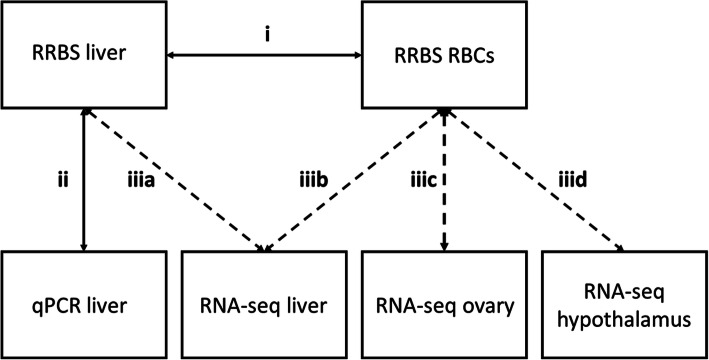
Overview of the data used in this study and how they were linked. Solid lines refer to associations in which only data from individual female great tits was used, while dashed lines refer to associations in which both individual (RRBS) and pooled (RNA-seq) data was used. Number-character combinations indicate the aims (see ‘Introduction’) of the study and the methods used (see ‘Methods’ for details)

CpG sites were annotated, using R packages ‘GenomicFeatures’ [49] and ‘rtracklayer’ [50], to different genomic locations: TSS region (300 bp upstream - 50 bp downstream of the annotated transcription start site), promoter region (2000 bp upstream - 200 bp downstream of the annotated transcription start site), gene body (exons and introns), and 10 kb up- and downstream regions (10 kb regions adjacent to the gene body, respectively). Each identified CpG site was assigned to one of the above specified genomic regions (and the gene annotated to that region) with BEDtools v.2.26.0 [51]. See Additional file 42; Table S23 for an overview on how many CpG sites were covered per genomic location in the RBCs and liver data and how many genes were associated to the CpG sites within a respective genomic region and tissue. Earlier studies in great tits have shown that methylation levels surrounding the TSS and within promoter regions best associate with RNA expression [21, 24]. Hence, only CpG sites in the TSS or promoter region of annotated genes were used for exploring (i) tissue-general and tissue-specific changes in DNA methylation between RBCs and liver and (ii) the correlation between change in methylation and candidate gene expression in liver (qPCR, see below) correlation. CpG sites within the TSS regions, promoter regions, gene body, and 10 kb up−/downstream regions were used for exploring (iii) genome-wide associations between changes in methylation and changes in gene expression in liver, hypothalamus, or ovary (Fig. 4).

### RNA extraction, real-time quantitative polymerase chain reaction and sequencing

From the same females, for which we acquired DNA methylation patterns, we used already available qPCR and RNA-seq data generated by two other studies [22, 39]. In short, RNA was isolated from hypothalamus, ovary and liver by Trizol extraction and reverse transcribed into cDNA [39].

#### qPCR

In a previous study [39] primer pairs were built based on the *Parus major* reference genome v1.1. and *Parus major* annotation release 101 (https://www.ncbi.nlm.nih.gov/genome/annotation_euk/Parus_major/101/) for a list of candidate genes with known and unknown functions in avian reproduction and checked for specificity using a BLAST search. Efficiency of each primer pair was determined by a 5-point standard curve of cDNA samples. Relative transcript levels were measured within hypothalamus, ovary and liver by real-time qPCR using the SYBR Green method followed by fluorescence measurements and analyses to obtain cycle thresholds. Expression levels of the candidate genes were normalized against reference genes. The combination and number of reference genes differ per organ and can be found elsewhere [39].

#### RNA sequencing

In a previous study, genome-wide expression patterns were measured in pools (*n* = 12) of three female great tits from both the early and late selection line [22]. This resulted in four pools per time point, of which every pool represented a selection line × treatment combination. Since the 18 females from the current study originate from the early selection line, we used the RNA-seq data from the pools with early selection line birds (*n* = 2 pools per time point with *n* = 3 females per pool). Briefly, libraries were prepared using the Illumina TruSeq strand-specific mRNA method (Illumina, San Diego, CA, USA) and one lane of Illumina HiSeq 2500 (single-end 50 bp) for 12 pools. Reads were filtered for low quality. Subsequently, trimmed reads were mapped to the *Parus major* reference genome v1.1, after which transcripts were assembled based on the *Parus major* annotation release 101. Unique reads that mapped to transcripts were counted.

### Statistical analysis

All statistics and plotting were performed using R version 3.5.2 [52]. An overview of how the different data sets and tissues are linked is provided in Fig. 4.

#### Tissue-general and tissue-specific changes in DNA methylation between red blood cells and liver

Prior to correlating the change in methylation over time between tissues, we tested whether methylation levels required standardization in order to meet the requirements to conduct such a correlation. For each sample we calculated the mean and variance in methylation proportion across all CpG sites within a sample and tested for a difference in mean and variance between samples with a Kruskal-Wallis test (*p* < 0.05, *n* = 18 for liver and RBCs, respectively) and Fligner Killeen test (*p* < 0.05, *n* = 18 for liver and RBCs, respectively), respectively (Additional file 43; Table S24). Because samples significantly differed in mean methylation and the variance of methylation proportion, we corrected the methylation proportion of individual CpG site for both differences. This was done by calculating z-scores; i.e. subtract the mean methylation proportion over all samples within the respective tissue from the methylation proportion of each individual CpG-site and divide this by the standard deviation of the methylation proportion over all samples within the respective tissue. We used the z-scores to calculated the change in CpG site methylation between time points within liver and RBCs.

We conducted a differential methylation analysis, using the ‘methylKit’ package [52], on the raw count data of 302,647 CpG sites that were common for both the blood and liver data, in order to find DMS between time point 1 and 2 (Δ_1,2_), and time point 2 and 3 (Δ_2,3_) in either blood or liver (tissue-specific change) or in both tissues (tissue-general change) (Additional file 9; Table S2, Additional file 10; Table S3). We considered a site significantly differentially methylated when the difference in methylation between time points ≥ 15% and a q-value ≤0.01.

We used the Pearson’s correlations coefficient (*r*) to evaluate the relationship between DNA methylation in blood and liver, as it measures linear trends. We repeated this for sites that were situated in the promoter and TSS regions of genes in both RBCs and liver.

Additionally, a gene ontology (GO) analysis was performed using the genes that carried the tissue-specific and tissue-general changing DMS to explore which functional groups (GO terms) are over-represented [53, 54] and possibly linked to timing of breeding. We divided the gene sets from the two time point comparisons into three groups for DMS located in the gene body, TSS region and/or promoter region. GO analysis was performed using Cytoscape plugin ClueGo 2.5.7 [55]. Using kappa statistics, ClueGo constructs and compares networks of GO terms. A two-sided hypergeometric test [56] was applied, Kappa score and network specificity were kept at default values. The GO term/pathway selection was at 5% and false discovery correction was performed using the Benjamini-Hochberg step-down method [57]. We used human (11.05.2020) gene ontologies and the Kyoto Encyclopedia of Genes and Genomes (KEGG) pathway [58] with three background gene set lists, which were all the genes covered by filtered CpG sites (15,103 genes), TSS regions covered by CpG sites (5731 genes) and promoter regions covered by CpG sites (9816 genes).

#### Correlation between change in methylation and candidate gene expression in liver

We selected those sites both in the TSS and promoter regions within genes in liver that were either key to reproductive functioning (i.e. in relation timing of breeding) or within the reference genes (i.e. to normalize qPCR expression data) (Additional files 44 and 45; Tables S25 and S26), and for which there was also qPCR gene expression data available [33, Additional file 46; Table S27]. In order to evaluate the association between DNA methylation changes and RNA expression changes in the TSS region, we found CpG sites with 10x coverage across all samples for five from the total candidate gene set analysed in liver [39]: beta-2-microglobulin (*B2M*), glucocorticoid receptor (*GR*), heat shock protein family B (small) member 1 (*HSPB1*), mineralocorticoid receptor (*MR*) and protein 2 kinase C alpha (*PRKCA*). In addition to these genes, two more genes, ribosomal protein 19 (*RPL19*) and succinate dehydrogenase complex flavoprotein subunit A (*SDHA*), could be evaluated for promoter regions. Per gene, we calculated Δ_1,2_ and Δ_2,3_ for both expression and methylation levels. For example, from the methylation level of an individual female in time point 2, methylation levels of all females in time point 1 (*n* = 6) were subtracted. Subsequently, these six values were used to calculate the average change in methylation per female in time point 1 across all females from time point 2, and vice versa (see Additional file 47; Fig. S20 for a visualization). The same process was repeated for the expression levels. Pearson’s correlations were used to evaluate relations between the average change in expression and average change in methylation levels. *P*-values were adjusted for multiple comparisons using the Benjamini-Hochberg procedure [57].

#### Genome-wide associations between change in methylation and gene expression

Here, we used RRBS data of individual females (*n* = 6 females per time point) and RNA-seq data of pools of the same females (*n* = 2 pools per time point with *n* = 3 females per pool) to relate changes in CpG site methylation to changes in expression of the associated gene. We examined how (a) the change in liver methylation related to the change in liver gene expression and how the change in RBC methylation related to gene expression change in (b) liver, (c) ovary, and (d) hypothalamus (Fig. 4). Four this, we used (a) 529,717 CpG sites in the liver that were located within 14,982 genes in the liver, (b) 297,916 CpG sites in RBCs that were located within 13,893 genes in liver, (c) 14,708 genes in the ovary, and/or (d) 14,570 genes in the hypothalamus. To associate DNA methylation changes with gene-expression changes, CpG sites that showed a time-point effect were identified using a differential methylation analysis with time-point (levels 1,2 and 3) as a fixed factor, and genes with a significant time effect were identified using a differential gene expression analysis performed in [22]. This in contrast to the analysis of i), where we assessed changes for both time point 1 and 2 (Δ_1,2_), and time point 2 and 3 (Δ_2,3_) separately. We therefore end up with different numbers of significant sites for these two analyses.

Differential methylation analyses were performed for 529,717 CpG sites in liver and 297,916 CpG sites in RBCs using the ‘methylKit’ package [52]. To test the significance of a time-point effect, we used a model comparison approach to test whether the full model, including time point and temperature environment as fixed factors, explained the methylation profile of a site better than the null model only including the temperature environment as fixed effect. We considered a time effect to be significant for sites with q-value ≤0.01 for both tissues. This likely leads to a more stringent correction for multiple testing in liver as the number of sites tested was much higher than for RBCs. Differential expression analysis is described in detail in [22]. In short, main effect models for time point and selection line were tested using the standard DeSeq2 protocol [59] and a likelihood ratio test such that the main effect models were compared to a model excluding the main effect. Models were performed separately for each tissue. Genes present in the trimmed RNA-seq data sets for liver, hypothalamus, and ovary with adjusted *p* < 0.05 when testing the main effect model for a time point effect and with adjusted *p >* 0.05 when testing the main effect model for a selection line effect (as data from both selection lines were included when testing the main effect models) based on [22], were classified as genes that significantly changed in expression over time. Thereafter, to examine the association between DNA methylation change and change in gene expression in tissue comparisons a-d, we quantified the change between time point 1 and 2 (Δ_1,2_) and time point 2 and 3 (Δ_2,3_) for both the methylation level of CpG sites and gene expression levels. We quantified the change in methylation level, by first calculating the average methylation levels (i.e. methylation proportion × 100) per CpG site across females for all three time points and then calculated the difference between the respective time points per CpG site. We quantified the change in gene expression between time point 1 and 2 (Δ_1,2_), and between time point 2 and 3 (Δ_2,3_) separately, by calculating the log_2_Foldchange contrast using DeSeq2 [59]. We furthermore trimmed the data sets by excluding CpG sites with a change in methylation level < 5% methylation (since absolute methylation levels are lower in TSS regions) and genes with a change in expression (as log_2_Foldchange) < 0.5 for any of the two time-point contrasts. To better understand the effect of the genomic location on the relationship between changes in DNA methylation and gene expression, we differentiated between genomic locations (i.e. TSS region, promoter region, gene body and 10 kb up−/downstream region, see section ‘Gene annotation’, above). For each combination of comparison (a-d), time contrast (first and second) and genomic location, we plotted the gene expression as log_2_Foldchange against the change in methylation level. There are four possible quadrants of association between change in gene expression and change in methylation level: hypo-methylation and increased gene expression (Q1), hyper-methylation and increased gene expression (Q2), hyper-methylation and decreased gene expression (Q3), and hypo-methylation and decreased gene expression (Q4). While Q1 and Q3 would relate to changes in the predicted directions (based on the expectation that methylation and expression are negatively correlated), Q2 and Q4 would relate to changes opposite to the predicted directions. For the correlation between change in RBC methylation and change in gene expression in ovary, we tested whether CpG sites that were situated in the TSS region were enriched in quadrants Q1 or Q3 compared to quadrants Q2 or Q4 using a Fisher’s exact test, in which we compared the proportion of associations within quadrants Q1 or Q3 between the TSS and 10 kb downstream region. We used the 10 kb downstream region as a control region for CpG sites randomly distributed across Q1-Q4 as we do not expect any relationship between DNA methylation changes and gene expression changes in this region [21, 24]. We did not use the 10 kb upstream region or gene body as control regions as the 10 kb upstream region overlaps with the promoter region (in which we would rather expect a relationship between methylation and gene expression) and at least parts of the gene body are hypothesized to show a relationship between methylation and gene expression.

## Supplementary Information

**Additional file 1: Figure S1.** Hierarchical clustering of RRBS samples (RBCs and liver).

**Additional file 2: Figure S2.** Principal component analysis of RRBS samples (RBCs and liver). Coloring by tissue (A), family (B), sampling time point (C), and temperature environment (D).

**Additional file 3: Figure S3.** Hierarchical clustering of RBC RRBS samples.

**Additional file 4: Figure S4.** Hierarchical clustering of liver RRBS samples.

**Additional file 5: Figure S5.** Principal component analysis of RBC RRBS samples. Coloring by family (A), sampling time point (B), and temperature environment (C).

**Additional file 6: Figure S6.** Principal component analysis of liver RRBS samples. Coloring by family (A), sampling time point (B), and temperature environment (C).

**Additional file 7: Figure S7.** Principal component analysis of RBC RRBS samples excluding the outlier sample displayed in Fig. S5. Coloring by family (A), sampling time point (B), and temperature environment (C).

**Additional file 8: Table S1.** CpG sites (*n* = 302,647) in both red blood cells and liver for which all females show ≥10x coverage.

**Additional file 9: Table S2.** CpG sites that showed a differential change between time point 1 and 2 in either RBCs, liver or in both tissues.

**Additional file 10: Table S3.** CpG sites that showed a differential change between time point 2 and 3 in either RBCs, liver or in both tissues.

**Additional file 11: Table S4.** Number of DMS in the set of CpG sites overlapping between liver and RBCs, in promoters and TSS, that showed a differential change between time point 1 and 2, and time point 2 and 3.

**Additional file 12: Table S5.** Genes covered by tissue-general and tissue-general changing DMS, or both.

**Additional file 13: Table S6.** GO terms found for genes associated with tissue-general and tissue-specific changing DMS, subdivided over gene regions and time points.

**Additional file 14: Table S7.** Number of CpG sites found within individual genes.

**Additional file 15: Table S8**. Correlations between the change in individual gene expression and change in methylation for both promoter and TSS regions. *P*-values were corrected (p-adjusted) with the Benjamini-Hochberg procedure.

**Additional file 16: Figure S8.** Mean (±s.e.) difference in both DNA methylation in promoter regions and RNA expression per female in time point 1 (in grey) across all females in time point 2, and vice versa (in black) for the individual genes.

**Additional file 17: Figure S9.** Mean (±s.e.) difference in both DNA methylation in promoter regions and RNA expression per female in time point 2 (in grey) across all females in time point 3, and vice versa (in black) for the individual genes.

**Additional file 18: Figure S10.** Mean (±s.e.) difference in both DNA methylation in TSS and RNA methylation per female in time point 1 (in grey) across all females in time point 2, and vice versa (in black) for the individual genes.

**Additional file 19: Figure S11.** Mean (±s.e.) difference in both DNA methylation in TSS and RNA methylation per female in time point 2 (in grey) across all females in time point 3, and vice versa (in black) for the individual genes.

**Additional file 20: Table S9.** CpG sites in liver with a significant change in methylation across time points.

**Additional file 21: Table S10.** CpG sites in RBCs with a significant change in methylation across time points.

**Additional file 22: Table S11.** Genes that significantly change across time points in hypothalamus.

**Additional file 23: Table S12.** Genes that significantly change across time points in ovary.

**Additional file 24: Table S13.** Genes that significantly change across time points in liver.

**Additional file 25: Figure S12.** Log2 fold change for the expression of genes in liver in relation to change in methylation level of a CpG site in liver within the 10 kb downstream region, gene body, promoter region, and 10 kb upstream region that gene for Δ_1,2_. Within the TSS region we did not find a significant change CpG site methylation located within a gene with significant change in expression. The four quadrants (see ‘Methods’) are separated by dotted lines and labelled as ‘Q1-Q4’. Transparency is applied to the grey data points such that the area of overlap of between data points appears darker.

**Additional file 26: Figure S13.** Log2 fold change for the expression of genes in liver in relation to change in methylation level of a CpG site in liver within the 10 kb downstream region, gene body, promoter region, TSS region, and 10 kb upstream region of that gene for Δ_2,3_. The four quadrants (see ‘Methods’) are separated by dotted lines and labelled as ‘Q1-Q4’. Transparency is applied to the grey data points such hat the area of overlap of between data points appears darker.

**Additional file 27: Figure S14.** Log2 fold change for the expression of genes in hypothalamus in relation to change in methylation level of a CpG site in red blood cells within the 10 kb downstream region and gene body of that gene Δ_1,2_. Within the 10 kb upstream region, promoter region, and TSS region we did not find a significant change CpG site methylation located within a gene with significant change in expression. The four quadrants (see ‘Methods’) are separated by dotted lines and labelled as ‘Q1-Q4’. Transparency is applied to the grey data points such that the area of overlap of between data points appears darker.

**Additional file 28: Figure S15.** Log2 fold change for the expression of genes in hypothalamus in relation to change in methylation level of a CpG site in red blood cells within the 10 kb downstream region, promoter region, TSS region, and 10 kb upstream region of that gene for Δ_2,3_. Within the gene body we did not find a significant change CpG site methylation located within a gene with significant change in expression. The four quadrants (see ‘Methods’) are separated by dotted lines and labelled as ‘Q1-Q4’. Transparency is applied to the grey data points such that the area of overlap of between data points appears darker.

**Additional file 29: Figure S16.** Log2 fold change for the expression of genes in ovary in relation to change in methylation level of a CpG site in red blood cells within the 10 kb downstream region, gene body, promoter region, TSS region, and 10 kb upstream region of that gene for Δ_1,2_. The four quadrants (see ‘Methods’) are separated by dotted lines and labelled as ‘Q1-Q4’. Transparency is applied to the grey data points such that the area of overlap of between data points appears darker.

**Additional file 30: Figure S17.** Log2 fold change for the expression of genes in ovary in relation to change in methylation level of a CpG site in red blood cells within the 10 kb downstream region, gene body, promoter region, TSS region, and 10 kb upstream region of that gene for Δ_2,3_. The four quadrants (see ‘Methods’) are separated by dotted lines and labelled as ‘Q1-Q4’. Transparency is applied to the grey data points such that the area of overlap of between data points appears darker.

**Additional file 31: Figure S18.** Log2 fold change for the expression of genes in liver in relation to change in methylation level of a CpG site in red blood cells within 10 kb downstream region, gene body, promoter region, TSS region, and 10 kb upstream region of that gene for Δ_1,2_. The four quadrants (see ‘Methods’) are separated by dotted lines and labelled as ‘Q1-Q4’. Transparency is applied to the grey data points such that the area of overlap of between data point appears darker.

**Additional file 32: Figure S19.** Log2 fold change for the expression of genes in liver in relation to change in methylation level of a CpG site in red blood cells within 10 kb downstream region, gene body, promoter region, TSS region, and 10 kb upstream region of that gene for Δ_2,3_. The four quadrants (see ‘Methods’) are separated by dotted lines and labelled as ‘Q1-Q4’. Transparency is applied to the grey data points such that the area of overlap of between data points appears darker.

**Additional file 33: Table S14.** Number of sites in liver changing in methylation in different gene regions between time point 1 and 2 within genes associated with either Q1 and Q3 or Q2 and Q4 in liver.

**Additional file 34: Table S15.** Number of sites in liver changing in methylation in different gene regions between time point 2 and 3 within genes associated with either Q1 and Q3 or Q2 and Q4 in liver.

**Additional file 35: Table S16.** Number of sites in RBC changing in methylation in different gene regions between time point 1 and 2 within genes associated with either Q1 and Q3 or Q2 and Q4 in hypothalamus.

**Additional file 36: Table S17.** Number of sites in RBC changing in methylation in different gene regions between time point 2 and 3 within genes associated with either Q1 and Q3 or Q2 and Q4 in hypothalamus.

**Additional file 37: Table S18.** Number of sites in RBC changing in methylation in different gene regions between time point 1 and 2 within genes associated with either Q1 and Q3 or Q2 and Q4 in ovary.

**Additional file 38: Table S19.** Number of sites in RBC changing in methylation in different gene regions between time point 2 and 3 within genes associated with either Q1 and Q3 or Q2 and Q4 in ovary.

**Additional file 39: Table S20.** Number of sites in RBC changing in methylation in different gene regions between time point 1 and 2 within genes associated with either Q1 and Q3 or Q2 and Q4 in liver.

**Additional file 40: Table S21.** Number of sites in RBC changing in methylation in different gene regions between time point 2 and 3 within genes associated with either Q1 and Q3 or Q2 and Q4 in liver.

**Additional file 41: Table S22.** Details on library preparation and sequencing.

**Additional file 42: Table S23.** Number of CpG sites per tissue (liver, red blood cells (RBCs)) and genomic location (see Methods Lines 545–550). Please note; genomic locations overlap such that a respective CpG sites can be present more than once. Number of genes refers to the total number of genes with (a) CpG site(s) in the respective genomic location and tissue.

**Additional file 43: Table S24.** Females differed significantly in mean and variance in methylation across all sites in both RBC and liver, as well as when considering only the promoter regions or TSS.

**Additional file 44: Table S25.** Methylation levels per CpG site for females in time point 1 and 2 within the genes of which qPCR data is available.

**Additional file 45: Table S26.** Methylation levels per sites for females in time point 2 and 3 within the genes of which qPCR data is available.

**Additional file 46: Table S27.** Expression levels of the individual genes from [39] for which all females show ≥10x coverage for associated CpG sites.

**Additional file 47: Figure S20.** Methodology to calculate, per individual gene, the change in methylation per site between time points by subtracting the CpG-site methylation level of a female in, for example, time point 2 with all females in time point 1. Subsequently the average change per female in time point 1 across all females from time point 2 is calculated, and vice versa. This procedure applies also to the change between time point 2 and 3 and expression levels (see ‘Methods’).

## Data Availability

The datasets supporting the conclusions of this article are available in the NCBI Sequence Read Archive for the RRBS and RNA-seq data (https://www.ncbi.nlm.nih.gov/sra, bioproject PRJNA208335, accession numbers SRR9644032-SRR9644067 for the quality trimmed reads and accession numbers SRX3209916-SRX320918 for the RRBS data) and the Dataverse repository for the qPCR data (https://dataverse.nl/dataset.xhtml?persistentId=hdl:10411/5CQPHI).

## Abbreviations

bp: Base pairs
CpG: Cytosine-phosphodiester bond-guanine
DMS: Differentially methylated site
GO: Gene ontology
PCR: Polymerase chain reaction
RBC: Red blood cell
RRBS: Reduced representation bisulfite sequencing
TSS: Transcription start site
