# Supplementary figures and images for: Temporal changes in DNA methylation and RNA expression in a small song bird: within- and between-tissue comparisons
Source: BMC Genomics. 2021 Jan 7;22:36. doi: 10.1186/s12864-020-07329-9 (PMC7792223; doi:10.1186/s12864-020-07329-9)

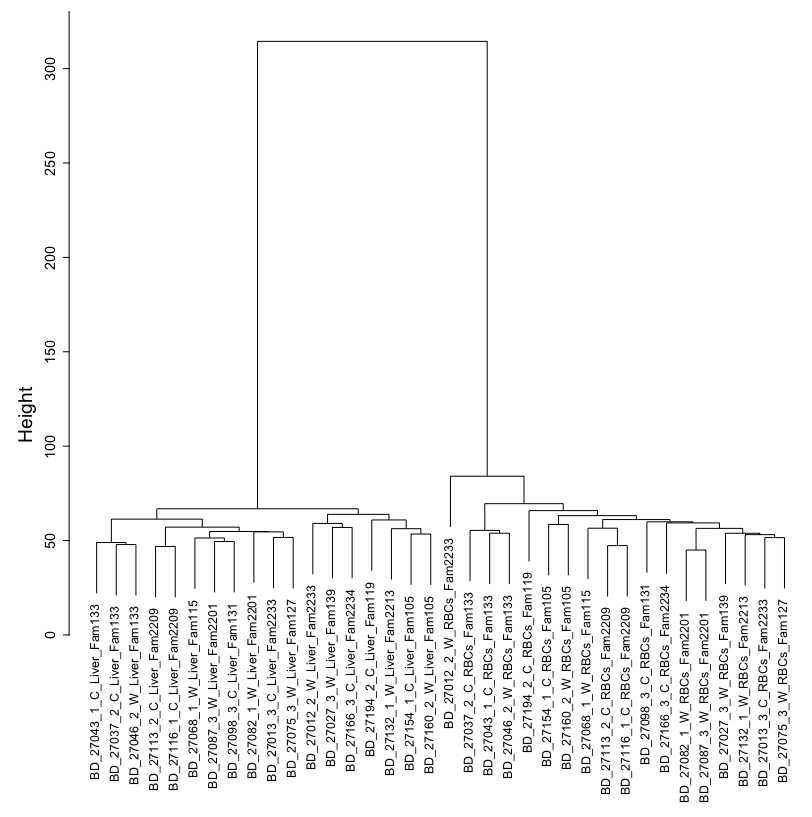

Supplement: Supplementary file 1 — Additional file 1: Figure S1. Hierarchical clustering of RRBS samples (RBCs and liver). [file 12864_2020_7329_MOESM1_ESM.tiff]

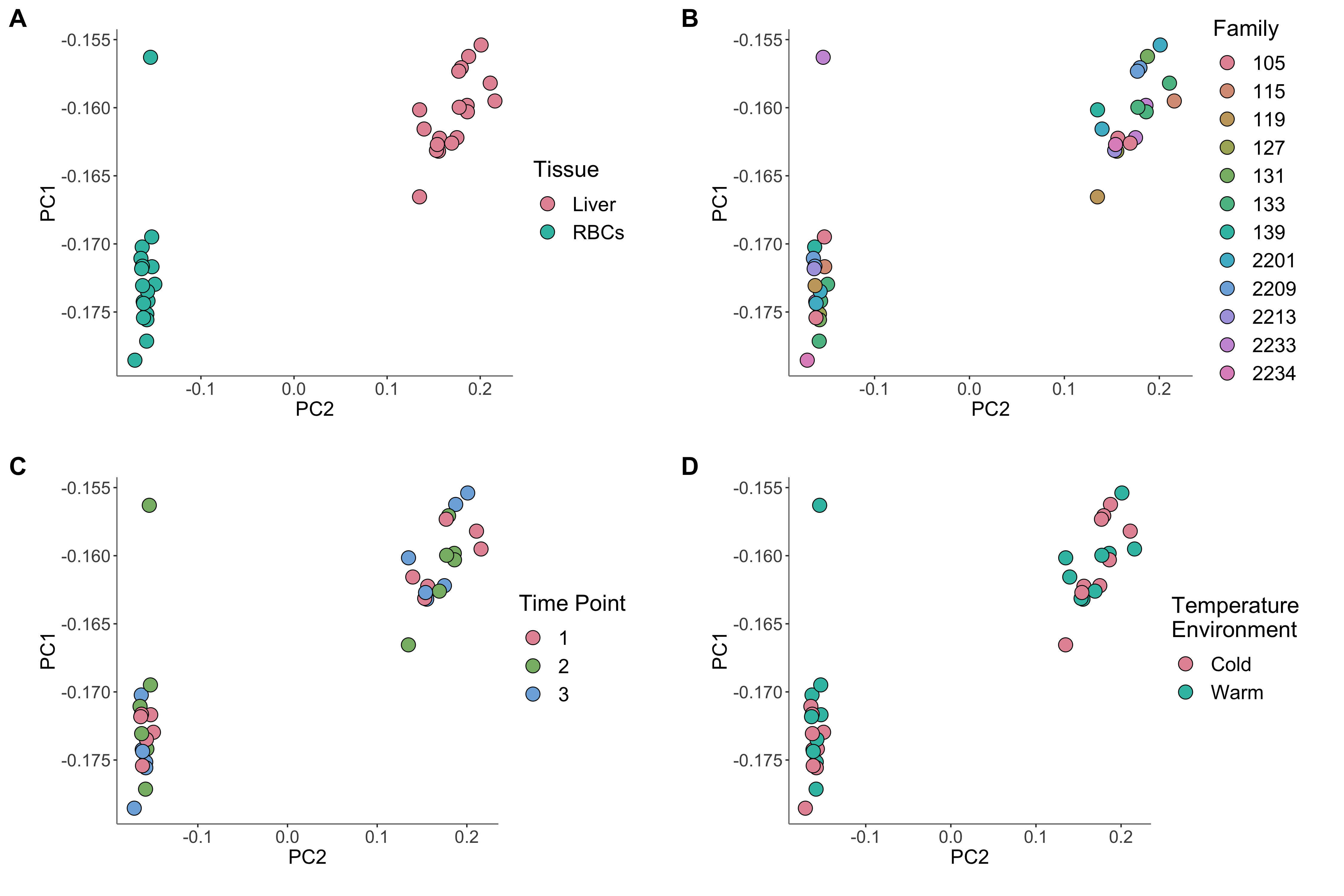

Supplement: Supplementary file 2 — Additional file 2: Figure S2. Principal component analysis of RRBS samples (RBCs and liver). Coloring by tissue (A), family (B), sampling time point (C), and temperature environment (D). [file 12864_2020_7329_MOESM2_ESM.tiff]

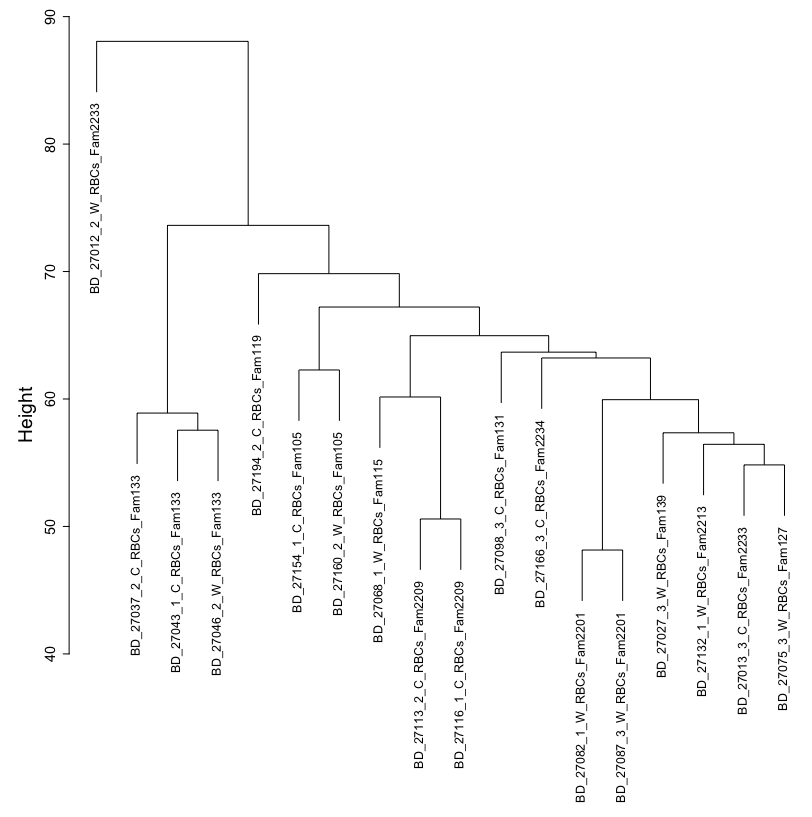

Supplement: Supplementary file 3 — Additional file 3: Figure S3. Hierarchical clustering of RBC RRBS samples. [file 12864_2020_7329_MOESM3_ESM.tiff]

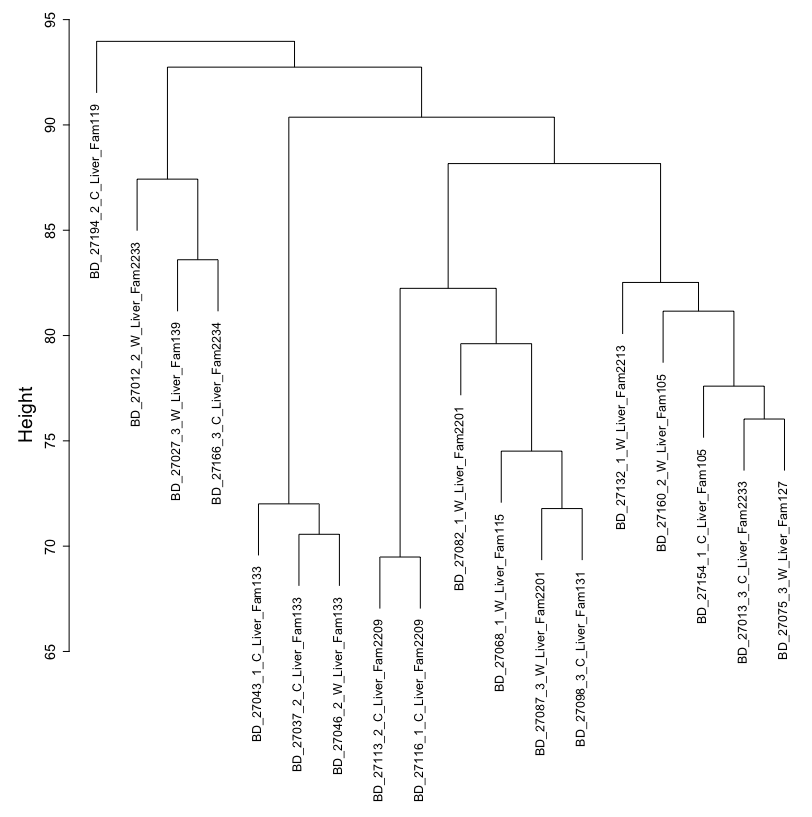

Supplement: Supplementary file 4 — Additional file 4: Figure S4. Hierarchical clustering of liver RRBS samples. [file 12864_2020_7329_MOESM4_ESM.tiff]

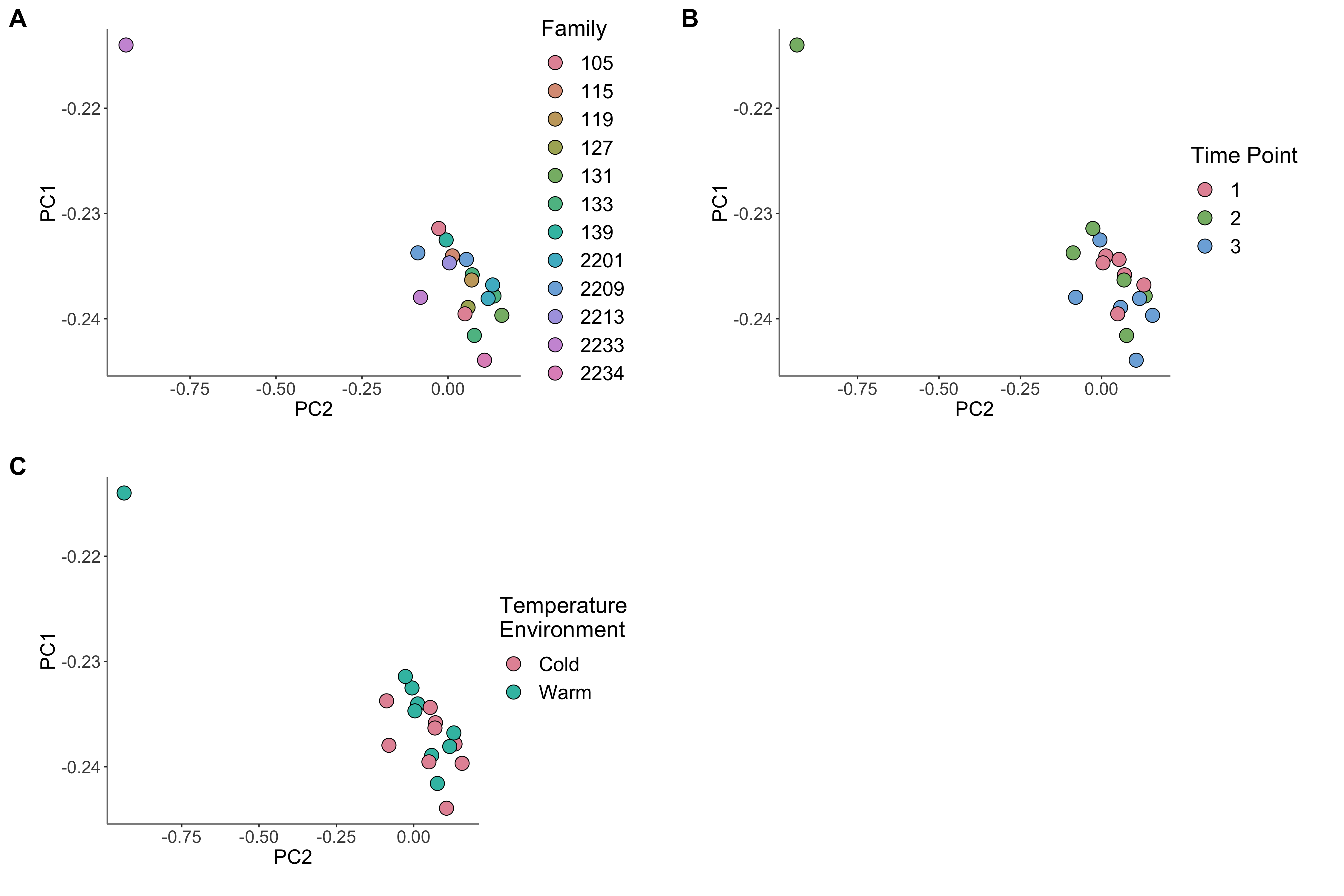

Supplement: Supplementary file 5 — Additional file 5: Figure S5. Principal component analysis of RBC RRBS samples. Coloring by family (A), sampling time point (B), and temperature environment (C). [file 12864_2020_7329_MOESM5_ESM.tiff]

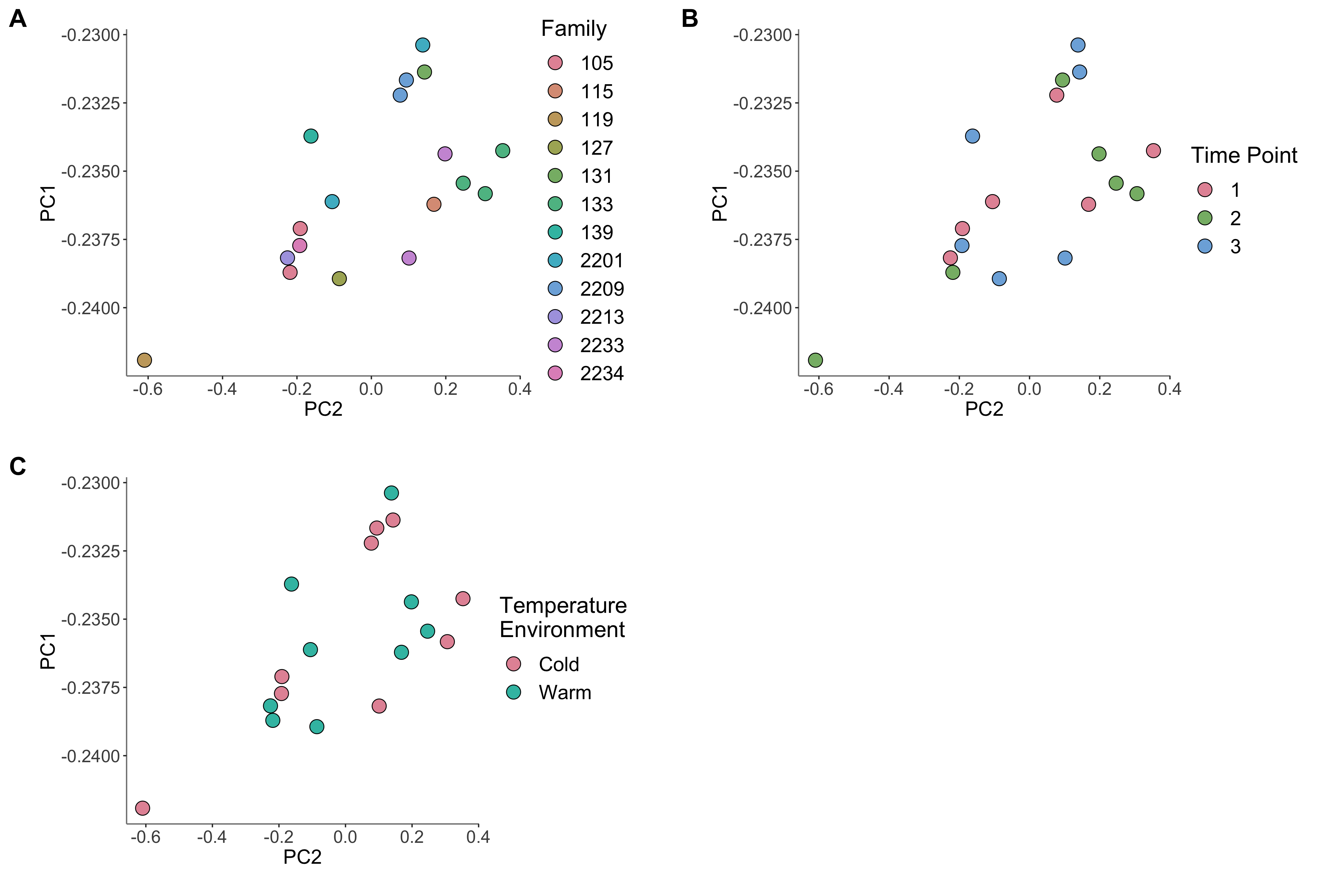

Supplement: Supplementary file 6 — Additional file 6: Figure S6. Principal component analysis of liver RRBS samples. Coloring by family (A), sampling time point (B), and temperature environment (C). [file 12864_2020_7329_MOESM6_ESM.tiff]

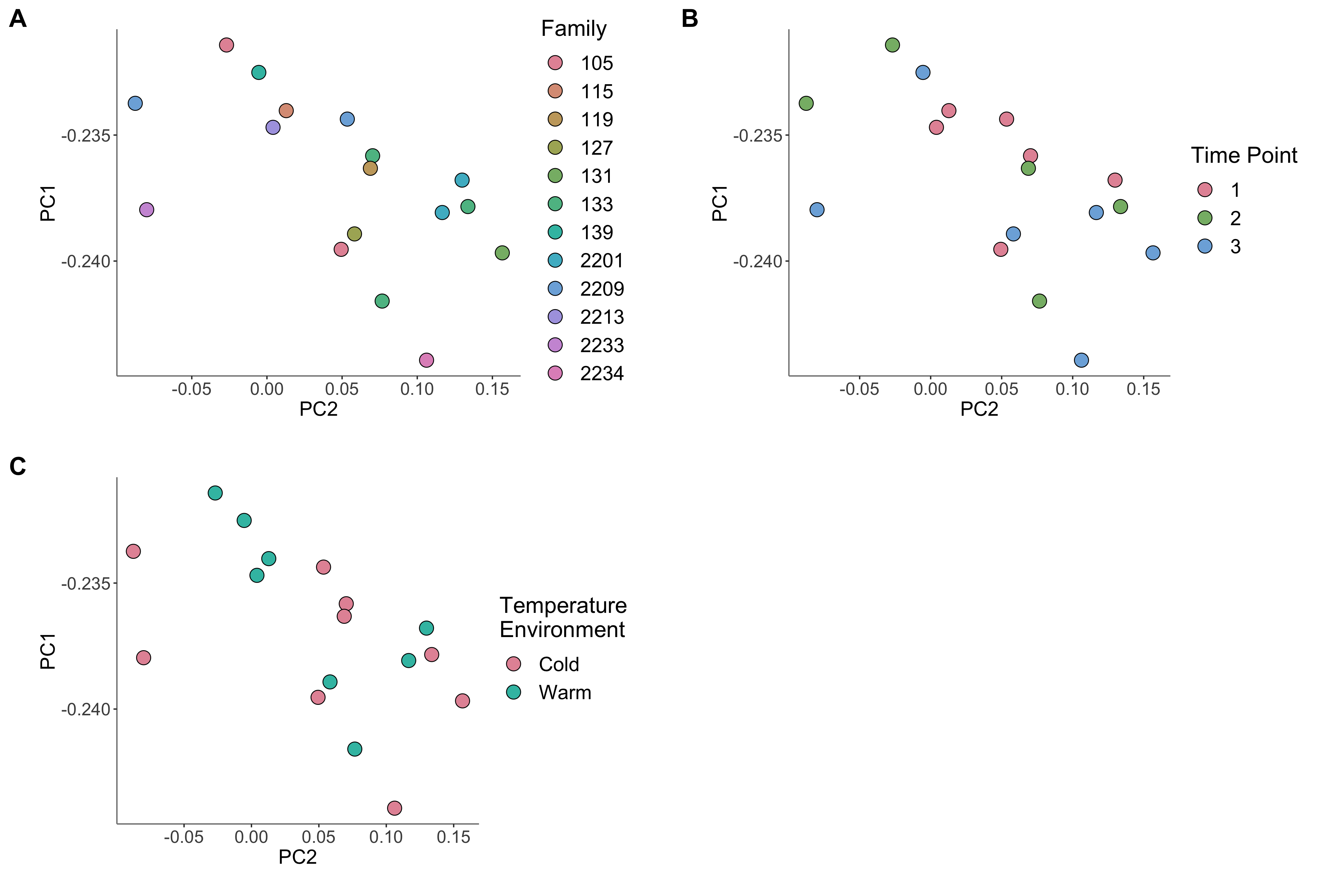

Supplement: Supplementary file 7 — Additional file 7: Figure S7. Principal component analysis of RBC RRBS samples excluding the outlier sample displayed in Fig. S5. Coloring by family (A), sampling time point (B), and temperature environment (C). [file 12864_2020_7329_MOESM7_ESM.tiff]

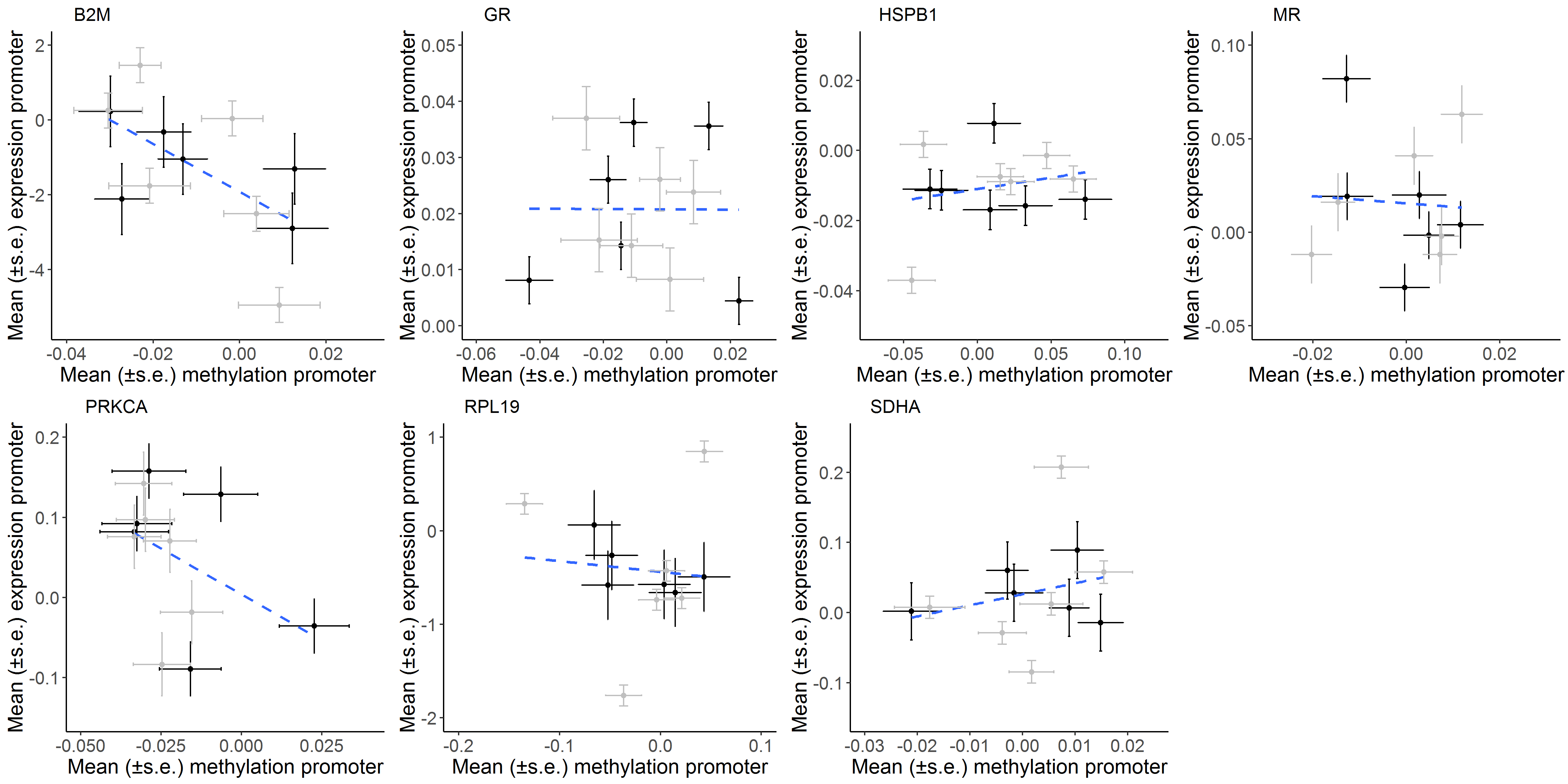

Supplement: Supplementary file 16 — Additional file 16: Figure S8. Mean (±s.e.) difference in both DNA methylation in promoter regions and RNA expression per female in time point 1 (in grey) across all females in time point 2, and vice versa (in black) for the individual genes. [file 12864_2020_7329_MOESM16_ESM.tiff]

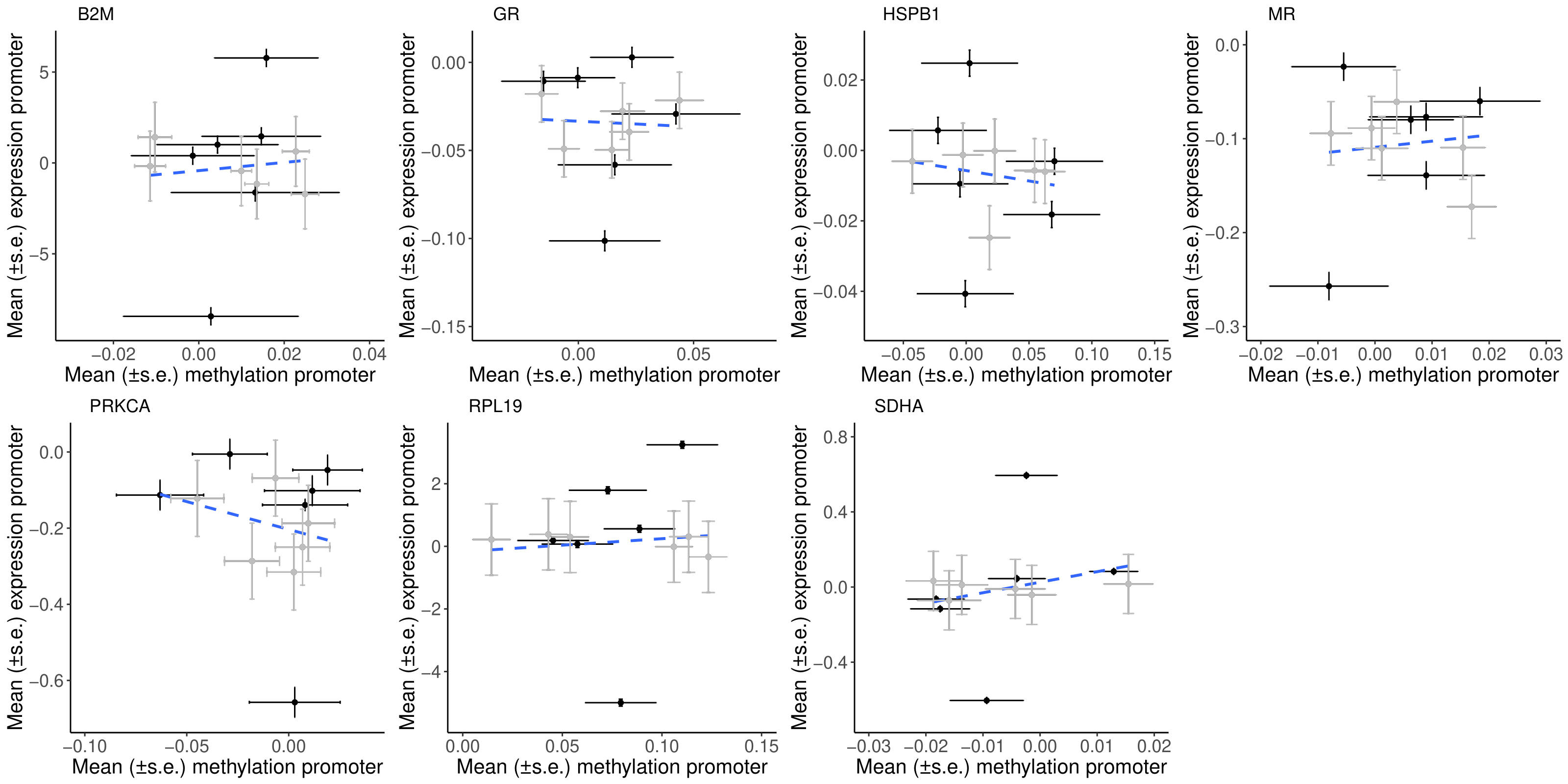

Supplement: Supplementary file 17 — Additional file 17: Figure S9. Mean (±s.e.) difference in both DNA methylation in promoter regions and RNA expression per female in time point 2 (in grey) across all females in time point 3, and vice versa (in black) for the individual genes. [file 12864_2020_7329_MOESM17_ESM.tiff]

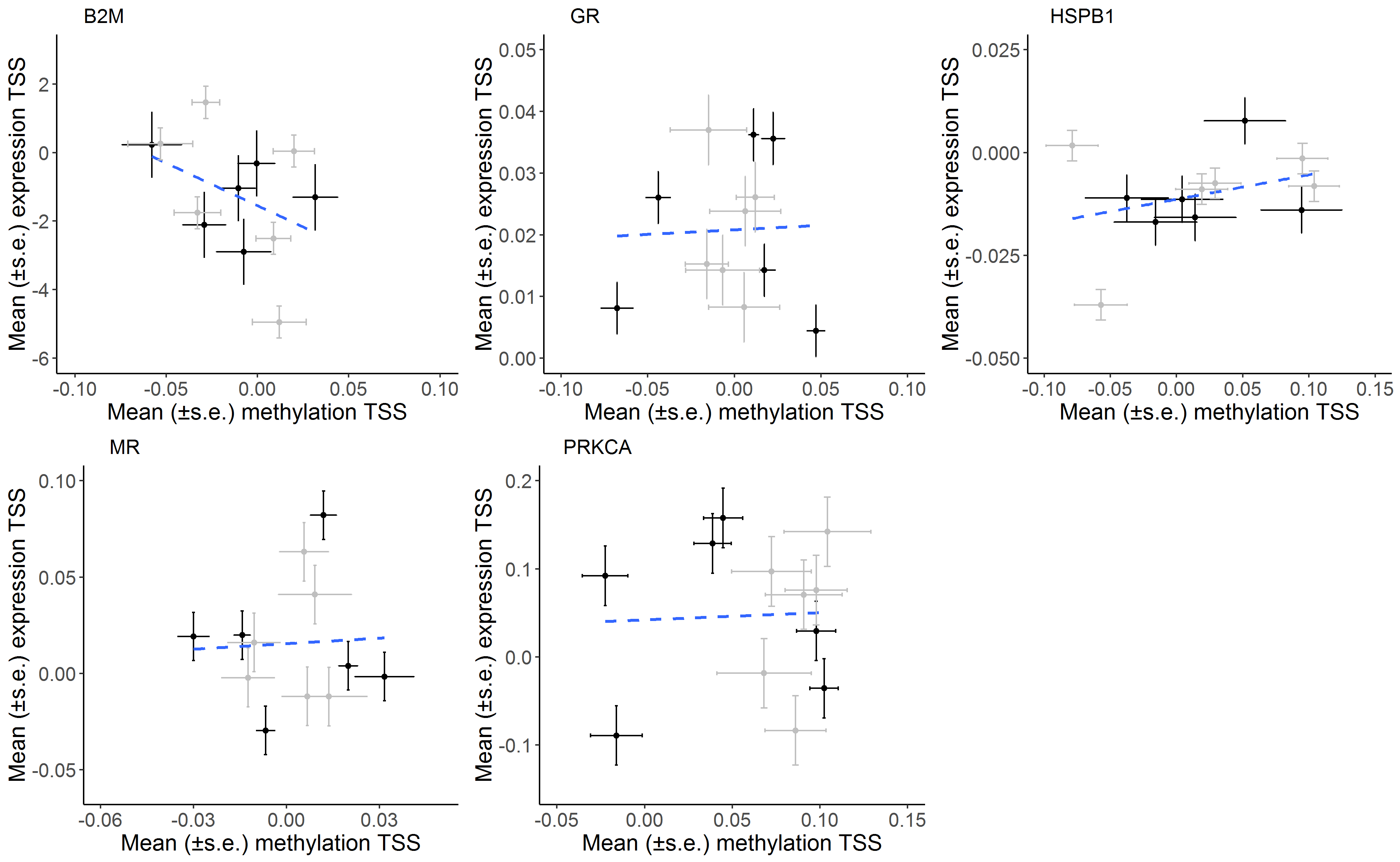

Supplement: Supplementary file 18 — Additional file 18: Figure S10. Mean (±s.e.) difference in both DNA methylation in TSS and RNA methylation per female in time point 1 (in grey) across all females in time point 2, and vice versa (in black) for the individual genes. [file 12864_2020_7329_MOESM18_ESM.tiff]

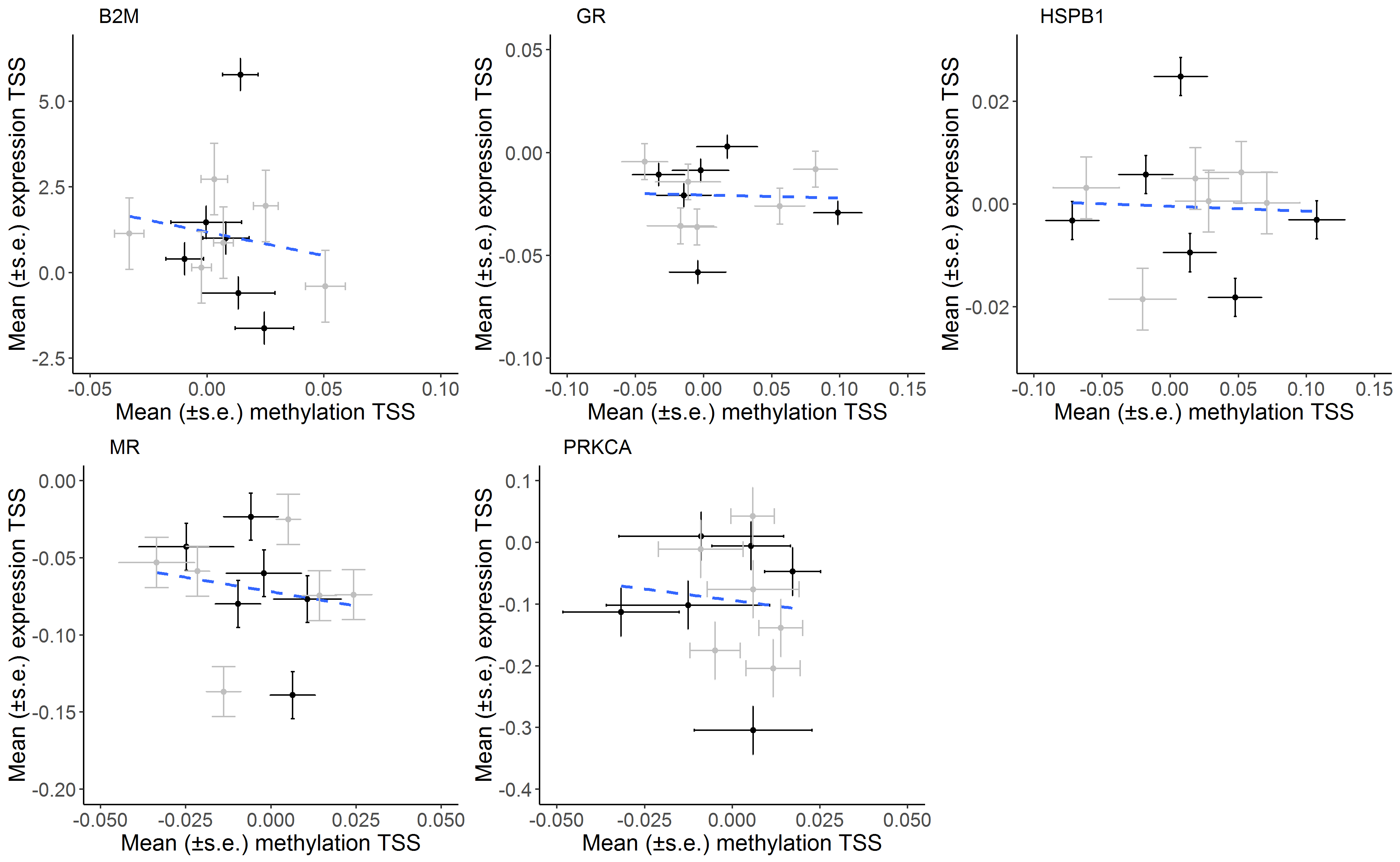

Supplement: Supplementary file 19 — Additional file 19: Figure S11. Mean (±s.e.) difference in both DNA methylation in TSS and RNA methylation per female in time point 2 (in grey) across all females in time point 3, and vice versa (in black) for the individual genes. [file 12864_2020_7329_MOESM19_ESM.tiff]

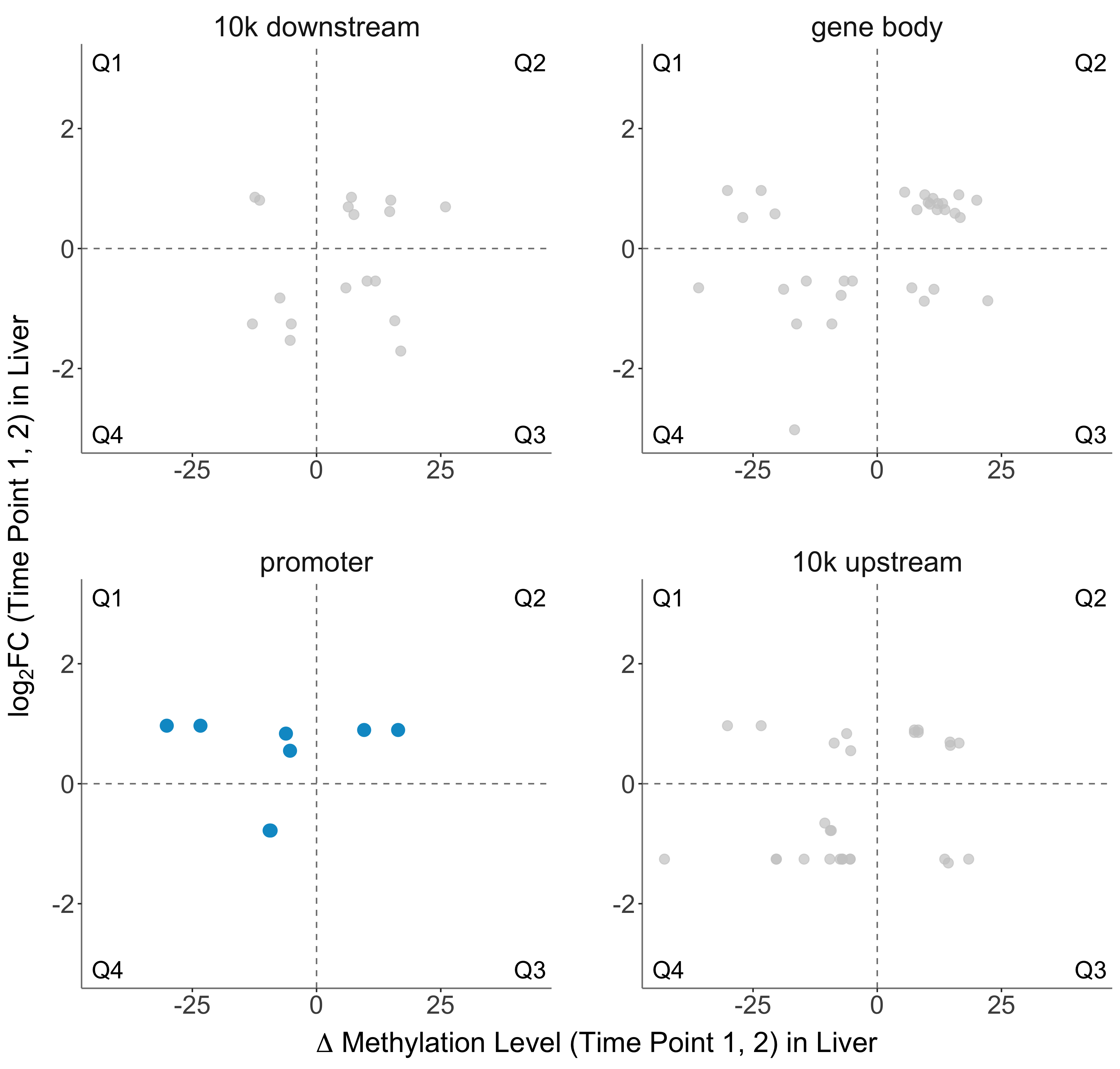

Supplement: Supplementary file 25 — Additional file 25: Figure S12. Log2 fold change for the expression of genes in liver in relation to change in methylation level of a CpG site in liver within the 10 kb downstream region, gene body, promoter region, and 10 kb upstream region that gene for Δ1,2. Within the TSS region we did not find a significant change CpG site methylation located within a gene with significant change in expression. The four quadrants (see ‘Methods’) are separated by dotted lines and labelled as ‘Q1-Q4’. Transparency is applied to the grey data points such that the area of overlap of between data points appears darker. [file 12864_2020_7329_MOESM25_ESM.tif]

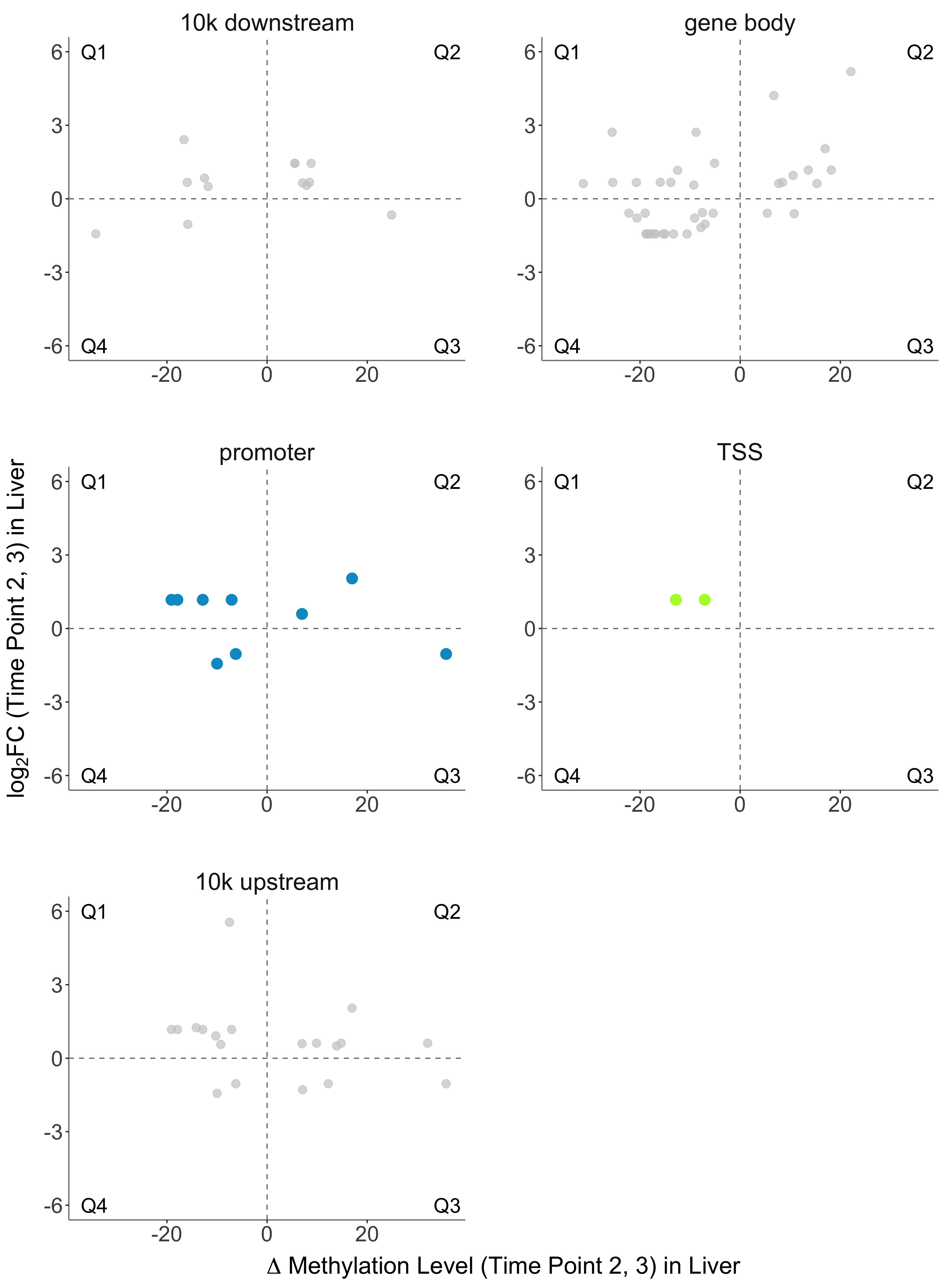

Supplement: Supplementary file 26 — Additional file 26: Figure S13. Log2 fold change for the expression of genes in liver in relation to change in methylation level of a CpG site in liver within the 10 kb downstream region, gene body, promoter region, TSS region, and 10 kb upstream region of that gene for Δ2,3. The four quadrants (see ‘Methods’) are separated by dotted lines and labelled as ‘Q1-Q4’. Transparency is applied to the grey data points such hat the area of overlap of between data points appears darker. [file 12864_2020_7329_MOESM26_ESM.tif]

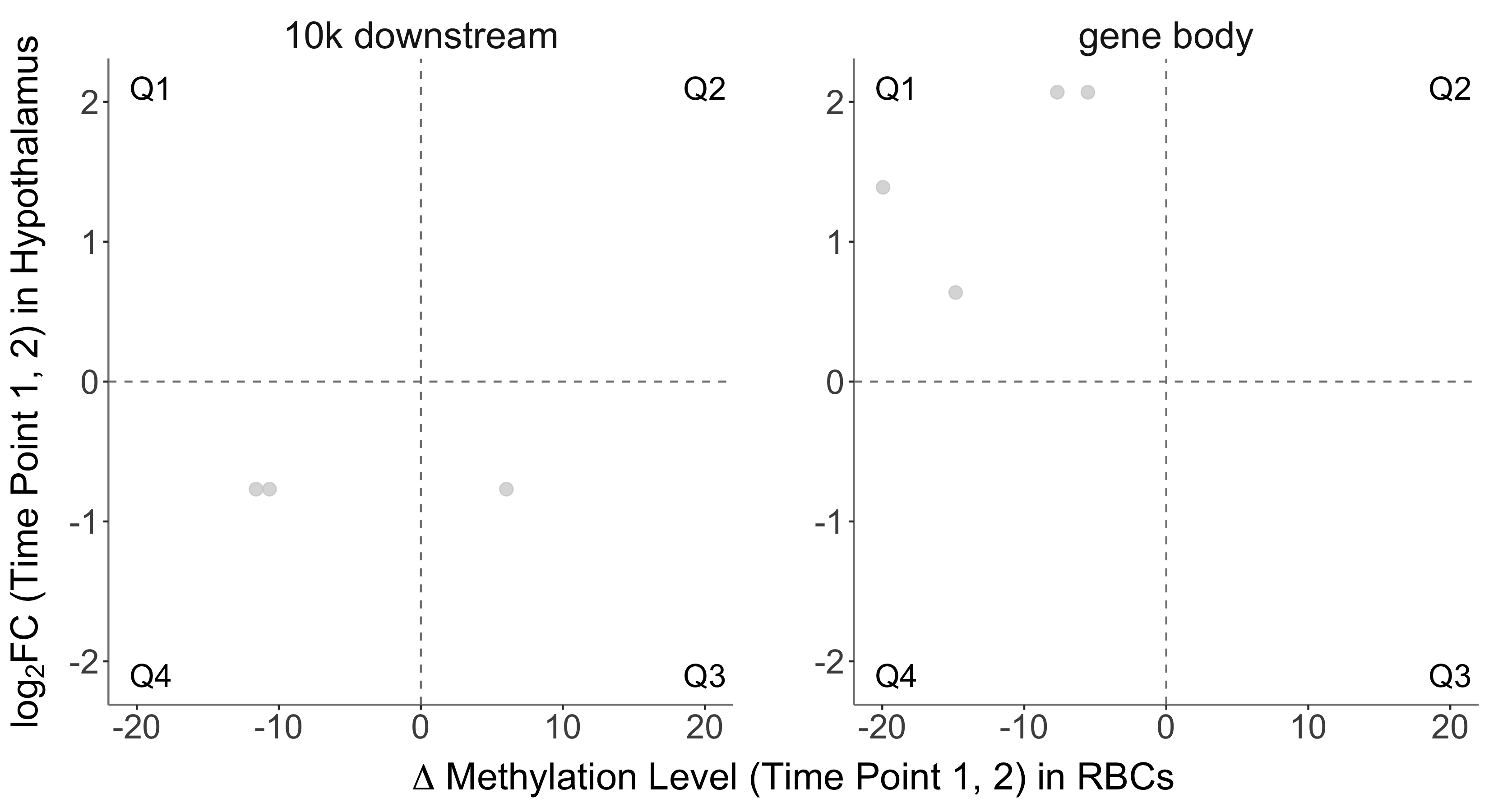

Supplement: Supplementary file 27 — Additional file 27: Figure S14. Log2 fold change for the expression of genes in hypothalamus in relation to change in methylation level of a CpG site in red blood cells within the 10 kb downstream region and gene body of that gene Δ1,2. Within the 10 kb upstream region, promoter region, and TSS region we did not find a significant change CpG site methylation located within a gene with significant change in expression. The four quadrants (see ‘Methods’) are separated by dotted lines and labelled as ‘Q1-Q4’. Transparency is applied to the grey data points such that the area of overlap of between data points appears darker. [file 12864_2020_7329_MOESM27_ESM.tif]

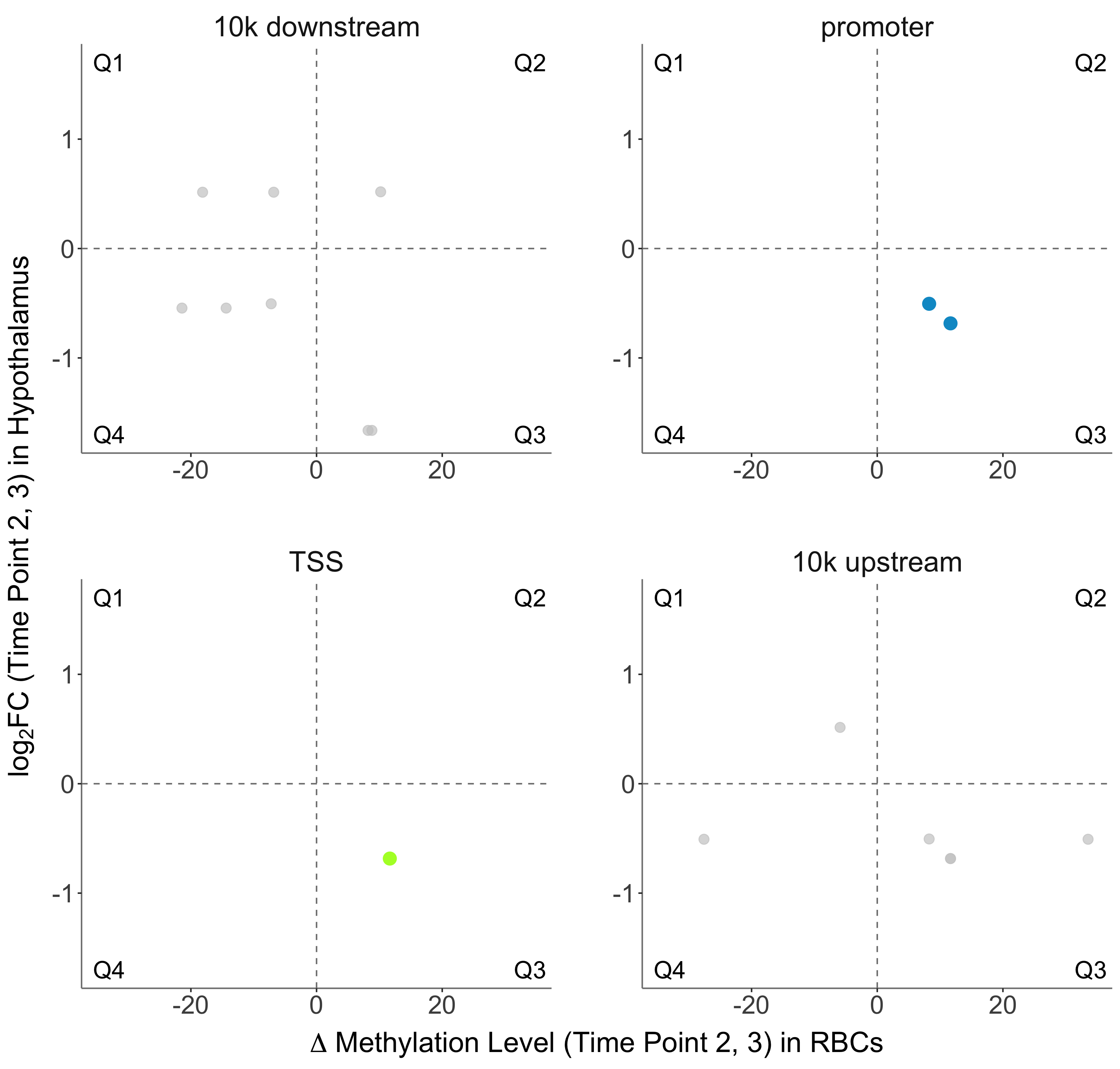

Supplement: Supplementary file 28 — Additional file 28: Figure S15. Log2 fold change for the expression of genes in hypothalamus in relation to change in methylation level of a CpG site in red blood cells within the 10 kb downstream region, promoter region, TSS region, and 10 kb upstream region of that gene for Δ2,3. Within the gene body we did not find a significant change CpG site methylation located within a gene with significant change in expression. The four quadrants (see ‘Methods’) are separated by dotted lines and labelled as ‘Q1-Q4’. Transparency is applied to the grey data points such that the area of overlap of between data points appears darker. [file 12864_2020_7329_MOESM28_ESM.tif]

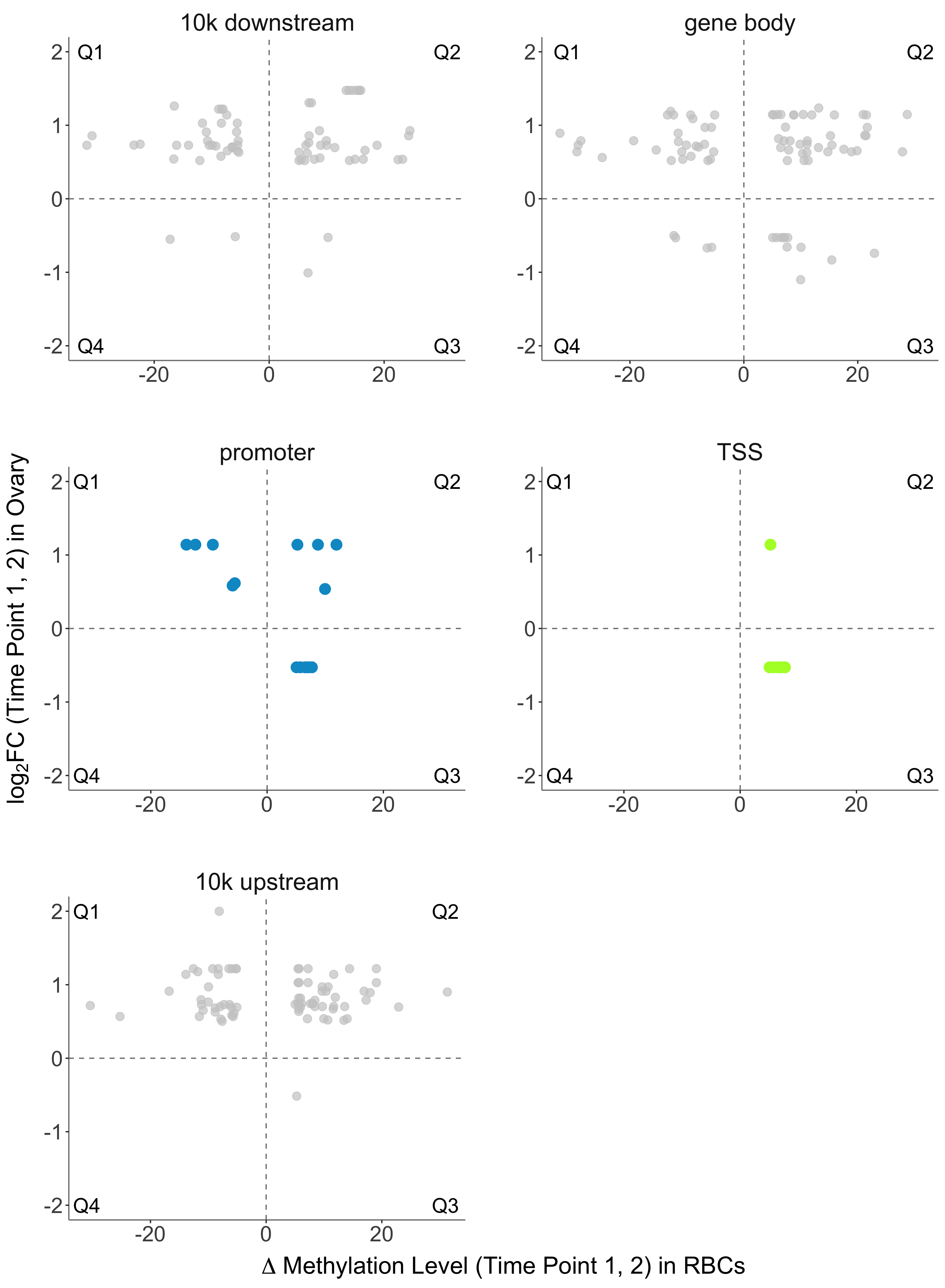

Supplement: Supplementary file 29 — Additional file 29: Figure S16. Log2 fold change for the expression of genes in ovary in relation to change in methylation level of a CpG site in red blood cells within the 10 kb downstream region, gene body, promoter region, TSS region, and 10 kb upstream region of that gene for Δ1,2. The four quadrants (see ‘Methods’) are separated by dotted lines and labelled as ‘Q1-Q4’. Transparency is applied to the grey data points such that the area of overlap of between data points appears darker. [file 12864_2020_7329_MOESM29_ESM.tif]

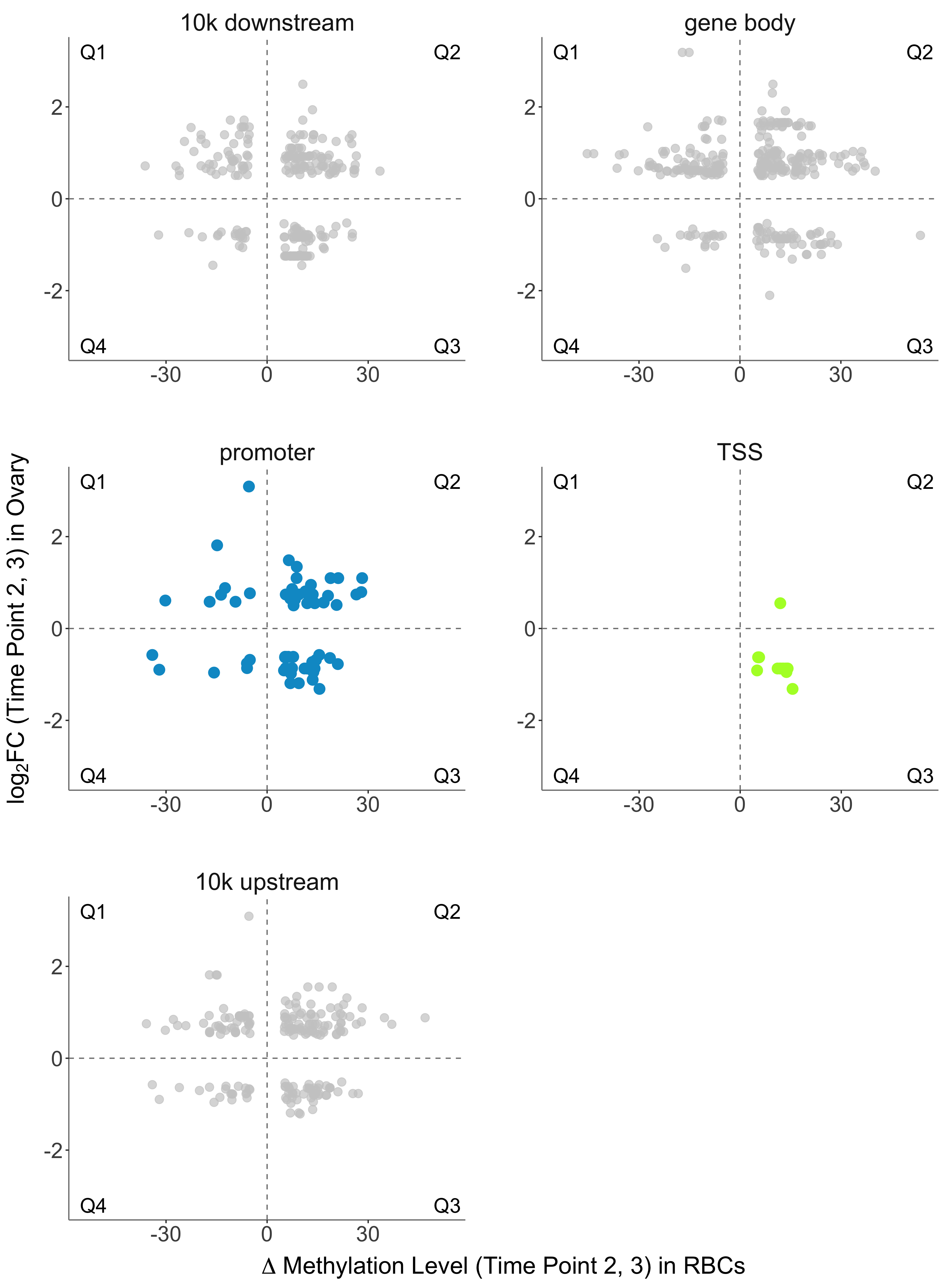

Supplement: Supplementary file 30 — Additional file 30: Figure S17. Log2 fold change for the expression of genes in ovary in relation to change in methylation level of a CpG site in red blood cells within the 10 kb downstream region, gene body, promoter region, TSS region, and 10 kb upstream region of that gene for Δ2,3. The four quadrants (see ‘Methods’) are separated by dotted lines and labelled as ‘Q1-Q4’. Transparency is applied to the grey data points such that the area of overlap of between data points appears darker. [file 12864_2020_7329_MOESM30_ESM.tif]

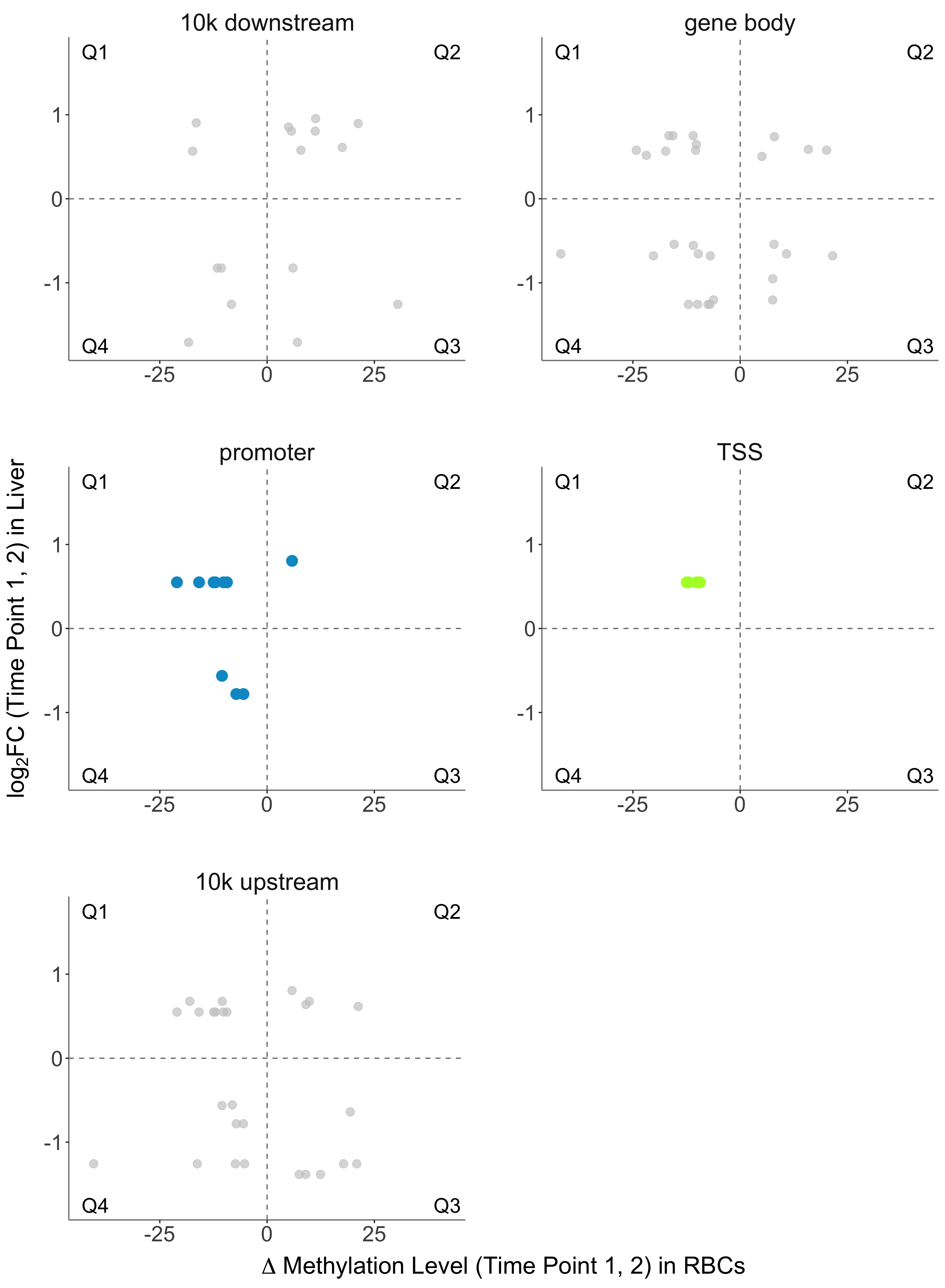

Supplement: Supplementary file 31 — Additional file 31: Figure S18. Log2 fold change for the expression of genes in liver in relation to change in methylation level of a CpG site in red blood cells within 10 kb downstream region, gene body, promoter region, TSS region, and 10 kb upstream region of that gene for Δ1,2. The four quadrants (see ‘Methods’) are separated by dotted lines and labelled as ‘Q1-Q4’. Transparency is applied to the grey data points such that the area of overlap of between data point appears darker. [file 12864_2020_7329_MOESM31_ESM.tif]

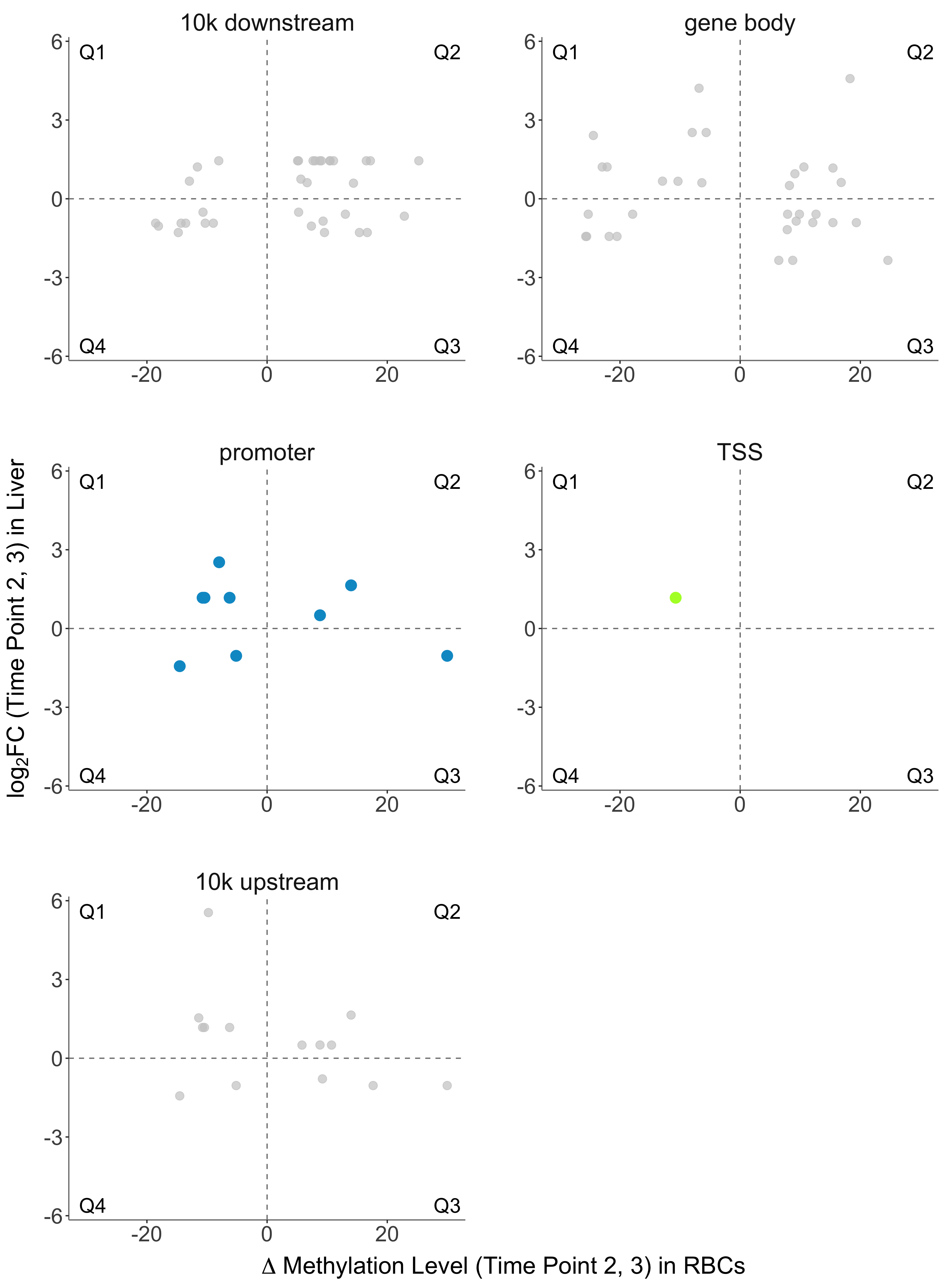

Supplement: Supplementary file 32 — Additional file 32: Figure S19. Log2 fold change for the expression of genes in liver in relation to change in methylation level of a CpG site in red blood cells within 10 kb downstream region, gene body, promoter region, TSS region, and 10 kb upstream region of that gene for Δ2,3. The four quadrants (see ‘Methods’) are separated by dotted lines and labelled as ‘Q1-Q4’. Transparency is applied to the grey data points such that the area of overlap of between data points appears darker. [file 12864_2020_7329_MOESM32_ESM.tif]

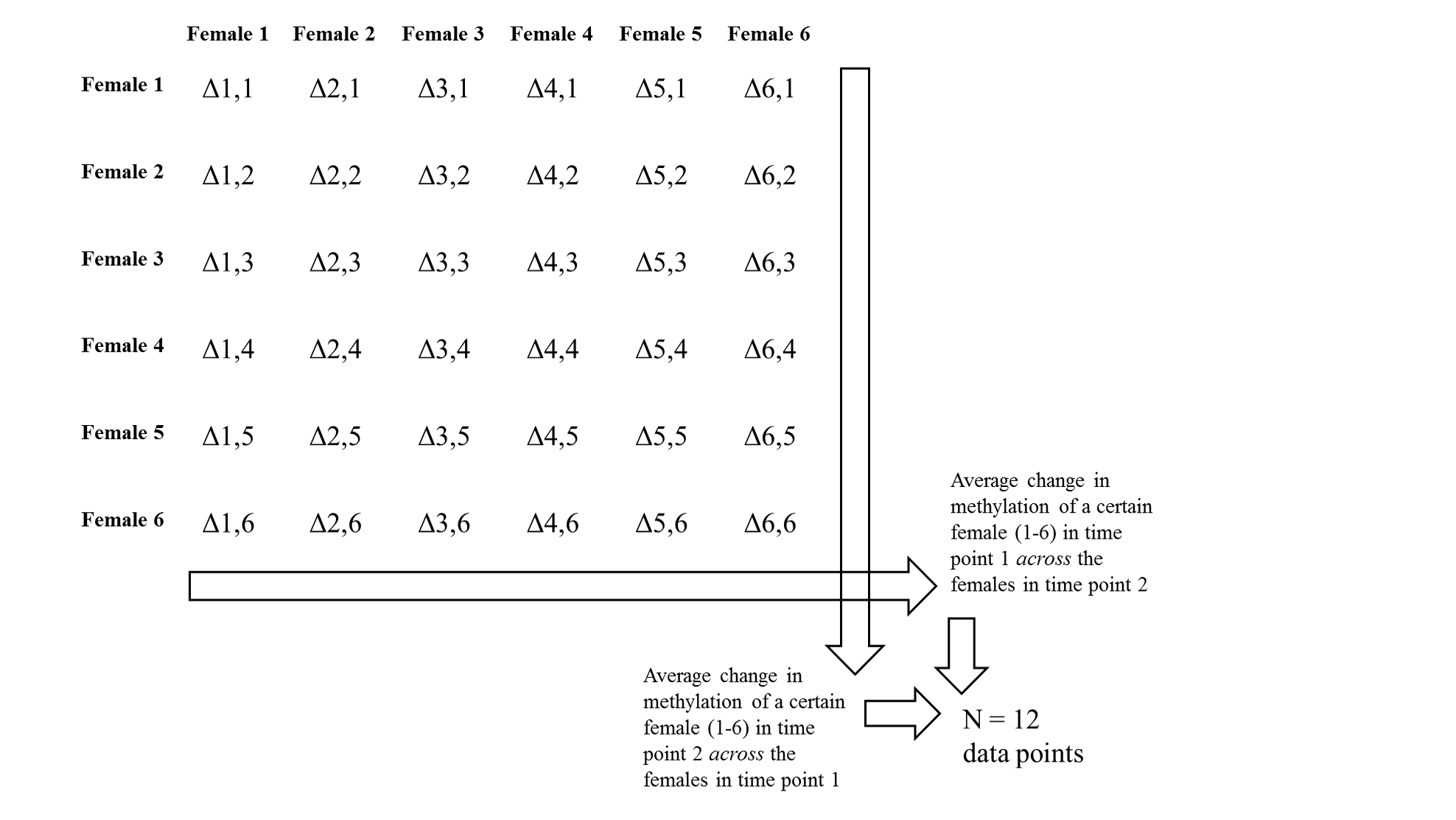

Supplement: Supplementary file 47 — Additional file 47: Figure S20. Methodology to calculate, per individual gene, the change in methylation per site between time points by subtracting the CpG-site methylation level of a female in, for example, time point 2 with all females in time point 1. Subsequently the average change per female in time point 1 across all females from time point 2 is calculated, and vice versa. This procedure applies also to the change between time point 2 and 3 and expression levels (see ‘Methods’). [file 12864_2020_7329_MOESM47_ESM.tif]
